# Supplementary material for: Contributing to Biochemistry and Optoelectronics: Pyrrolo[1′,2′:2,3]imidazo[1,5-a]indoles and Cyclohepta[4,5]pyrrolo[1,2-c]pyrrolo[1,2-a]imidazoles via [3+2] Annulation of Acylethynylcycloalka[b]pyrroles with Δ1-Pyrrolines
Source: Int J Mol Sci. 2023 Feb 8;24(4):3404. doi: 10.3390/ijms24043404 (PMC9959468; doi:10.3390/ijms24043404)
Supplement: Supplementary file 1 [file ijms-24-03404-s001.zip › ijms-2184969-supplementary.pdf]

Supporting Information

Contributing to biochemistry and optoelectronics: Pyrrolo[1’,2’:2,3]imidazo[1,5-*a*]indoles and cyclohepta[4,5]pyrrolo[1,2-*c*]pyrrolo[1,2-*a*]imidazoles *via* [3+2] annulation of acylethynylcycloalka[*b*]pyrroles with Δ^1-pyrrolines

Ludmila A. Oparina, Nikita A. Kolyvanov, Igor A. Ushakov, Lina P. Nikitina, Olga V. Petrova, Lyubov N. Sobenina, Konstantin B. Petrushenko, Boris A. Trofimov*

A.E. Favorsky Irkutsk Institute of Chemistry, Siberian Branch of the Russian Academy of Sciences
1 Favorsky Str., 664033 Irkutsk, Russia, Fax: +7 3952 419 346
E-mail: *boris_trofimov@irioch.irk.ru*

General Information. NMR spectra were recorded from solutions in $CDCl_3$ on Bruker DPX-400 and AV-400 spectrometers (Bruker, Billerica, MA, USA) (400.1 MHz for ^1H, 100.6 MHz for ^{13}C, and 40.5 MHz for ^{15}N). Chemical shifts (δ) were quoted in parts per million (ppm). The residual solvent peak, δ_H 7.27 and δ_C 77.10, was used as a reference. Coupling constants (J) were reported in Hertz (Hz). The abbreviations were used to express the multiplicates: s (singlet), d (doublet), dd (doublet of doublet), t (triplet), m (multiplet), nr (narrow), br (broad). The ^{15}N chemical shifts were referenced to CH_3NO_2. The configurational assignment and the substituent location for the compounds **5-7** are based on 2D (NOESY, ^1H-^{13}C HSQC, ^1H-^{13}C HMBC, ^1H-^{15}N HMBC) NMR spectroscopy data.
UV/Vis absorption spectra were measured on a Lambda-35 (Perkin-Elmer, Waltham, MA, USA) spectrophotometer. Fluorescence spectra were measured on a FLSP-920 combined steady state and time resolved fluorescence spectrometers (Edinburgh Instrument, Livingston, UK). All quantum chemistry calculations are carried out using the Gaussian 09. B.01 program package. The ground-state (S_0) geometry optimizations and the vertical excitations $S_0 \rightarrow S_i$ (i = 1−5) at the S_0 geometry were calculated at TD-CAMB3LYP method, with a split valence with polarization SVP basis set.

For labeling of H- and C-atoms for NMR assignments, see Figure.

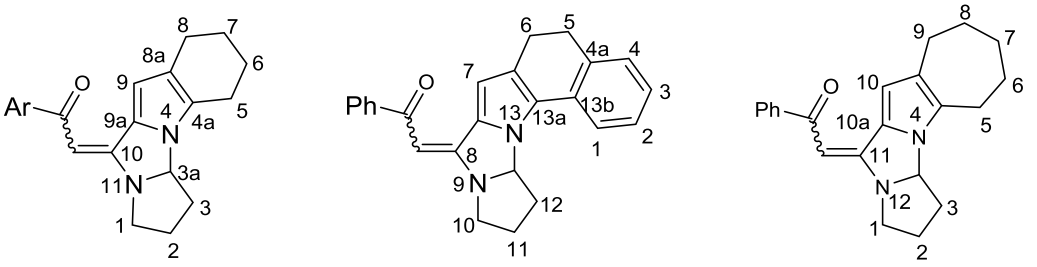

5 **6** **7**

Figure. Labeling of hydrogen and carbon atoms in compounds **5−7**.

IR spectra were obtained on a Varian 3100 IF-IR (Digilab LLC, USA) spectrometer (400-4000 cm^{-1}) as thin films dispersed from $CDCl_3$. Mass spectra of synthesized compounds were recorded on a GCMS-QP5050A spectrometer made by Shimadzu Company. High-resolution mass spectral analyses were performed from acetonitrile solution with 0.1% HFBA on HPLC Agilent 1200/Agilent 6210 TOF instrument equipped with an electrospray ionization (ESI) source (Agilent, USA). Melting points (uncorrected) were measured on a melting point apparatus SGW-X-4 (China). Thin layer chromatography was carried out on Merck silica gel 60 F254 pre-coated aluminium foil sheets and were visualized using UV light (254 nm). Column chromatography was carried out using slurry packed Alfa Aesar silica gel (SiO_2), 70-230 mesh, pore size 60 Å.

Copies of NMR and HRMS spectra of compounds 3, 5-7

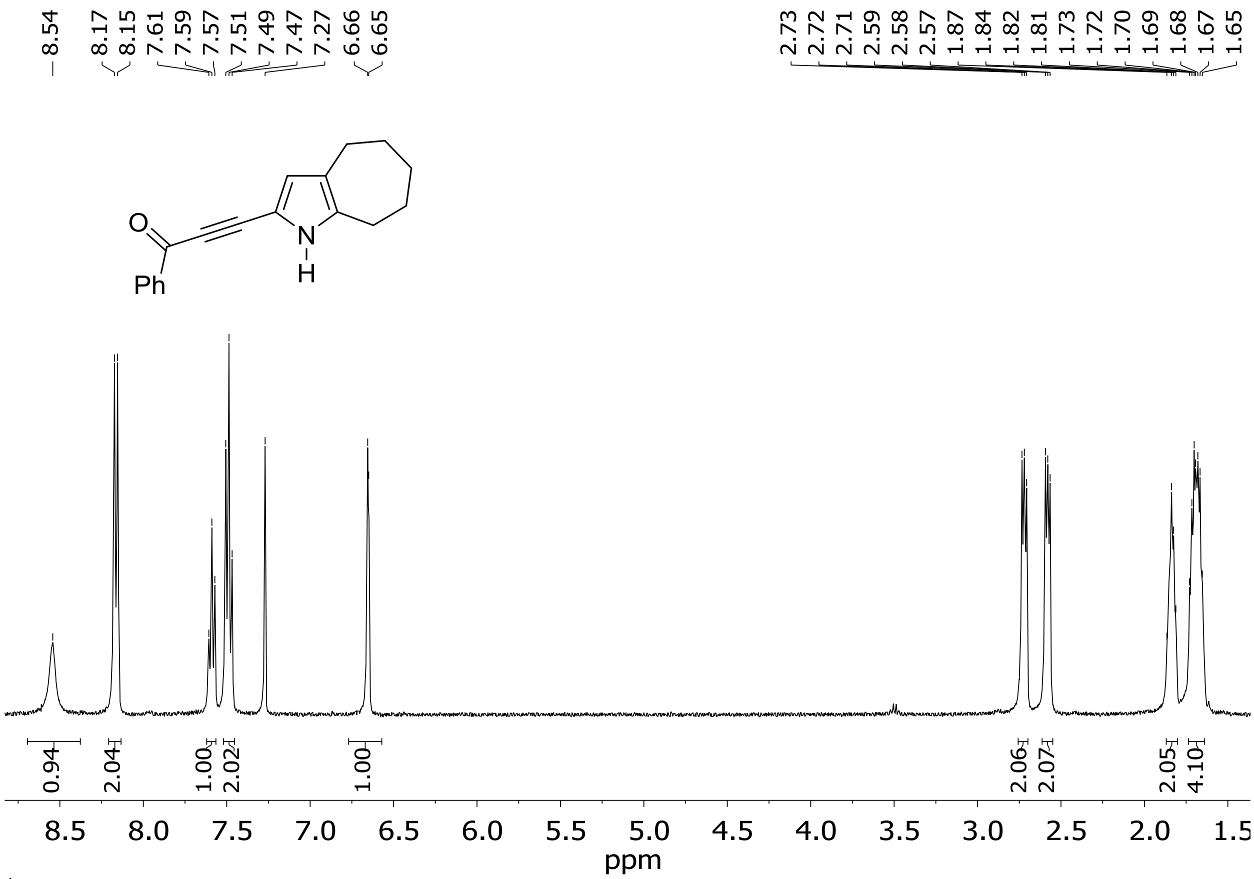

^{1}H NMR Spectrum of **3** (400.1 MHz, $CDCl_3$)

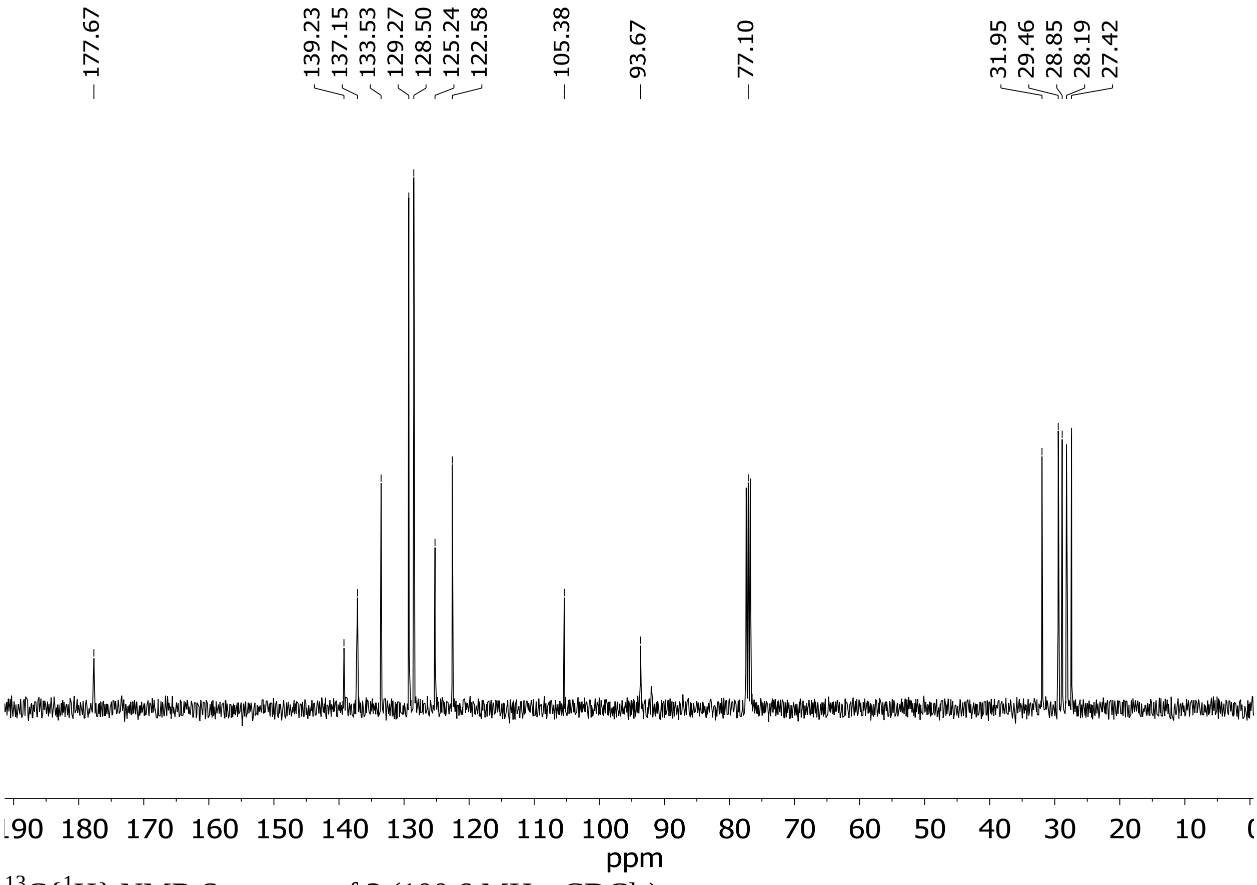

^{13}C{^{1}H} NMR Spectrum of **3** (100.6 MHz, $CDCl_3$)

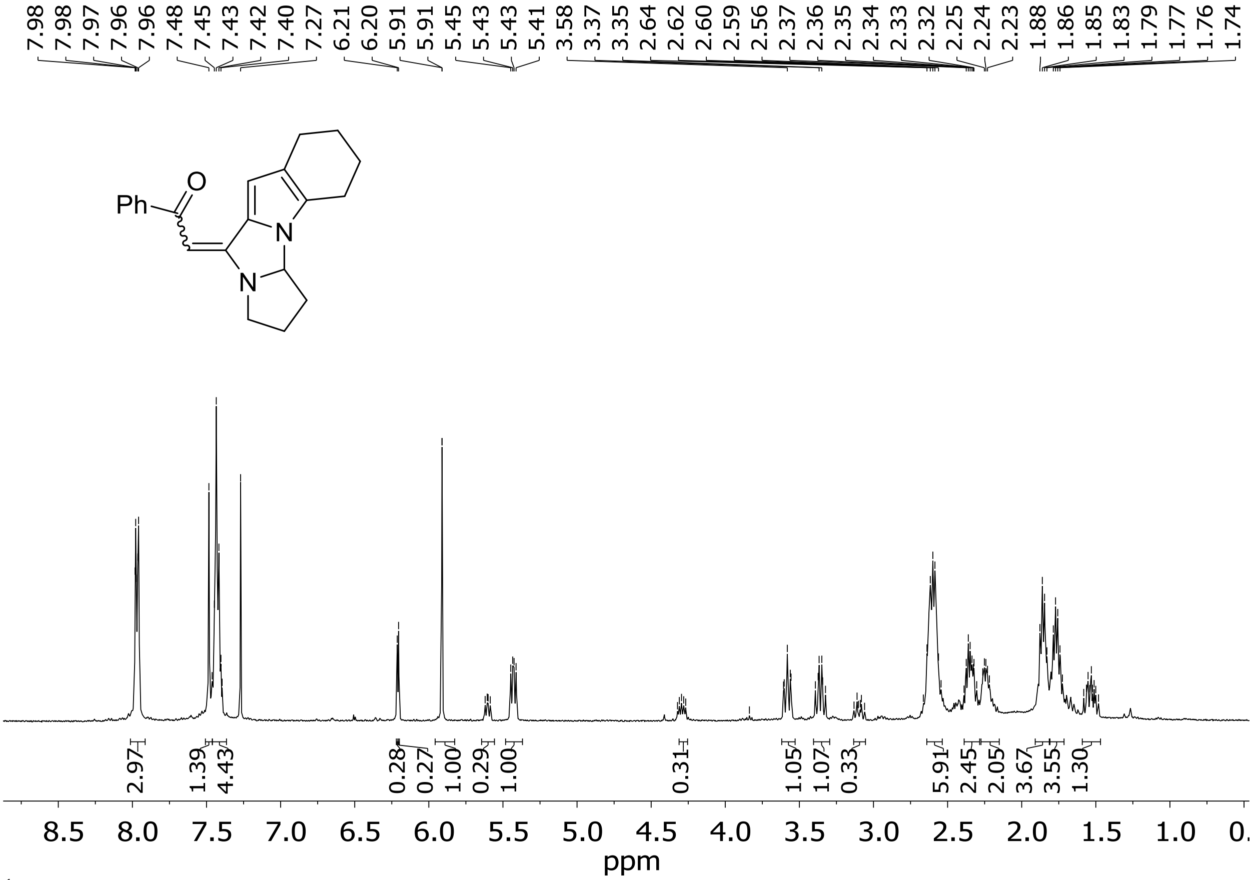

^{1}H NMR Spectrum of **5a** (400.1 MHz, $CDCl_3$)

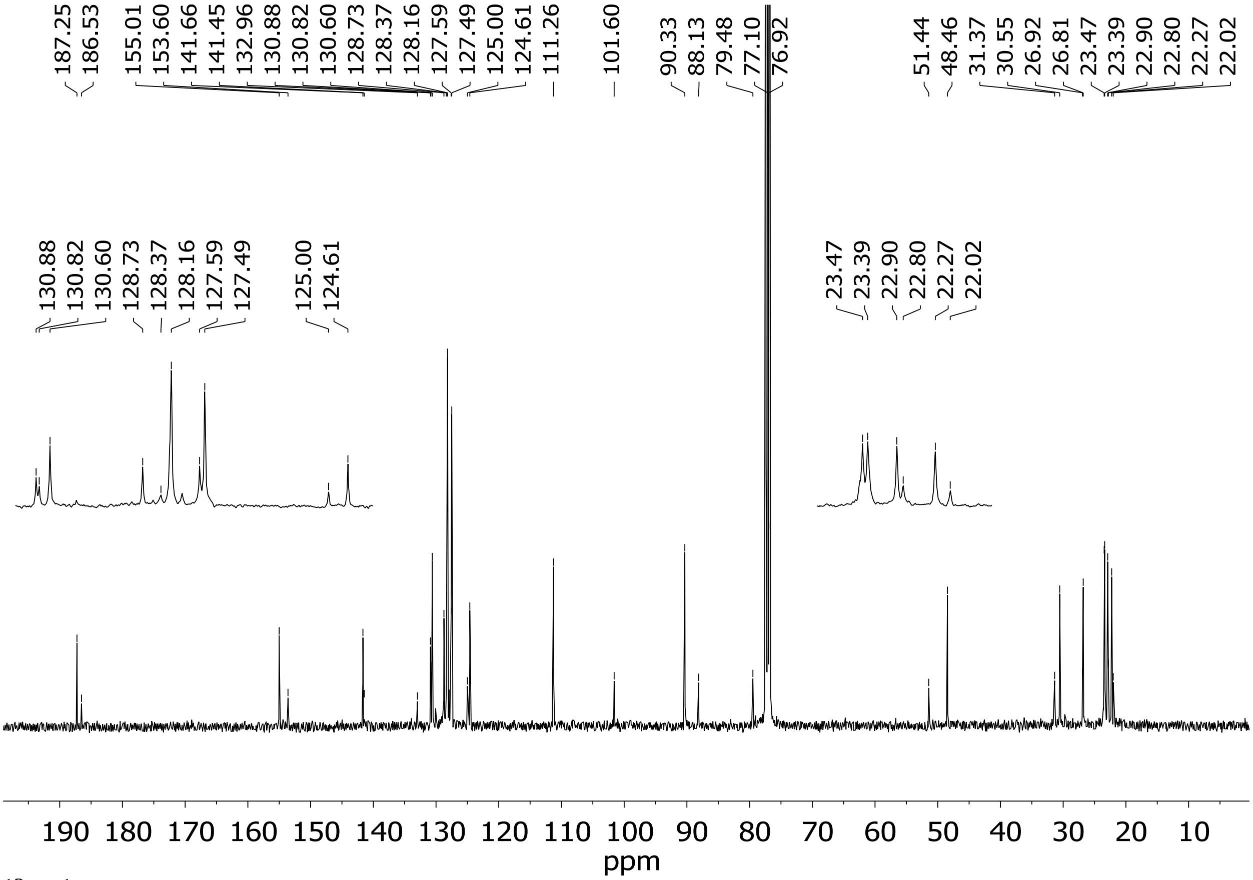

^{13}C{^{1}H} NMR Spectrum of **5a** (100.6 MHz, $CDCl_3$)

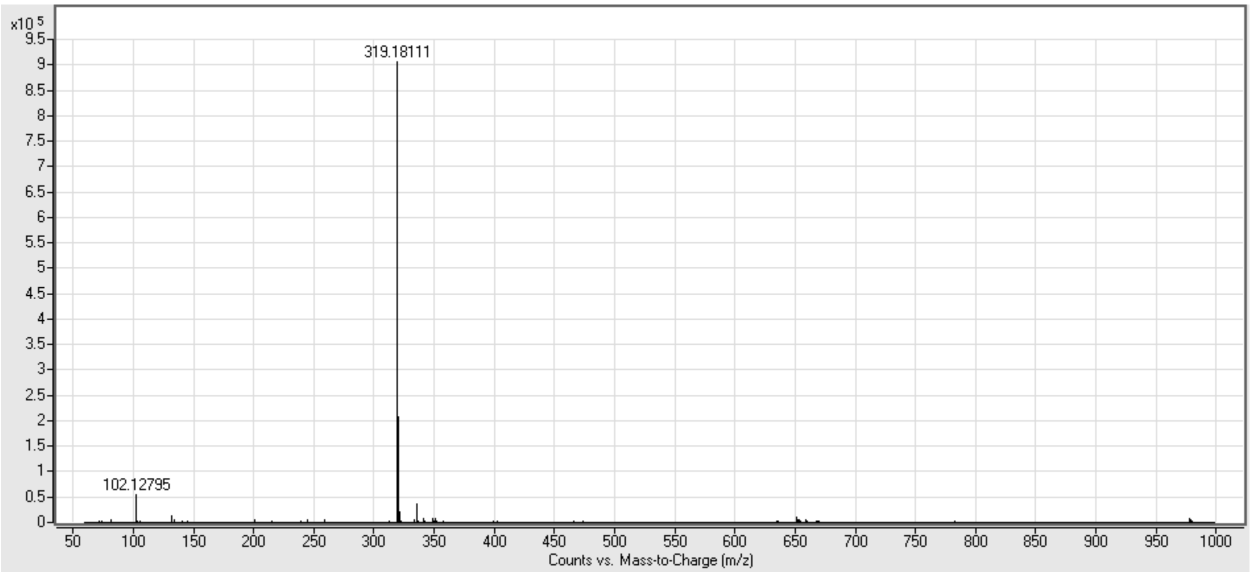

HRMS Spectrum of **5a**

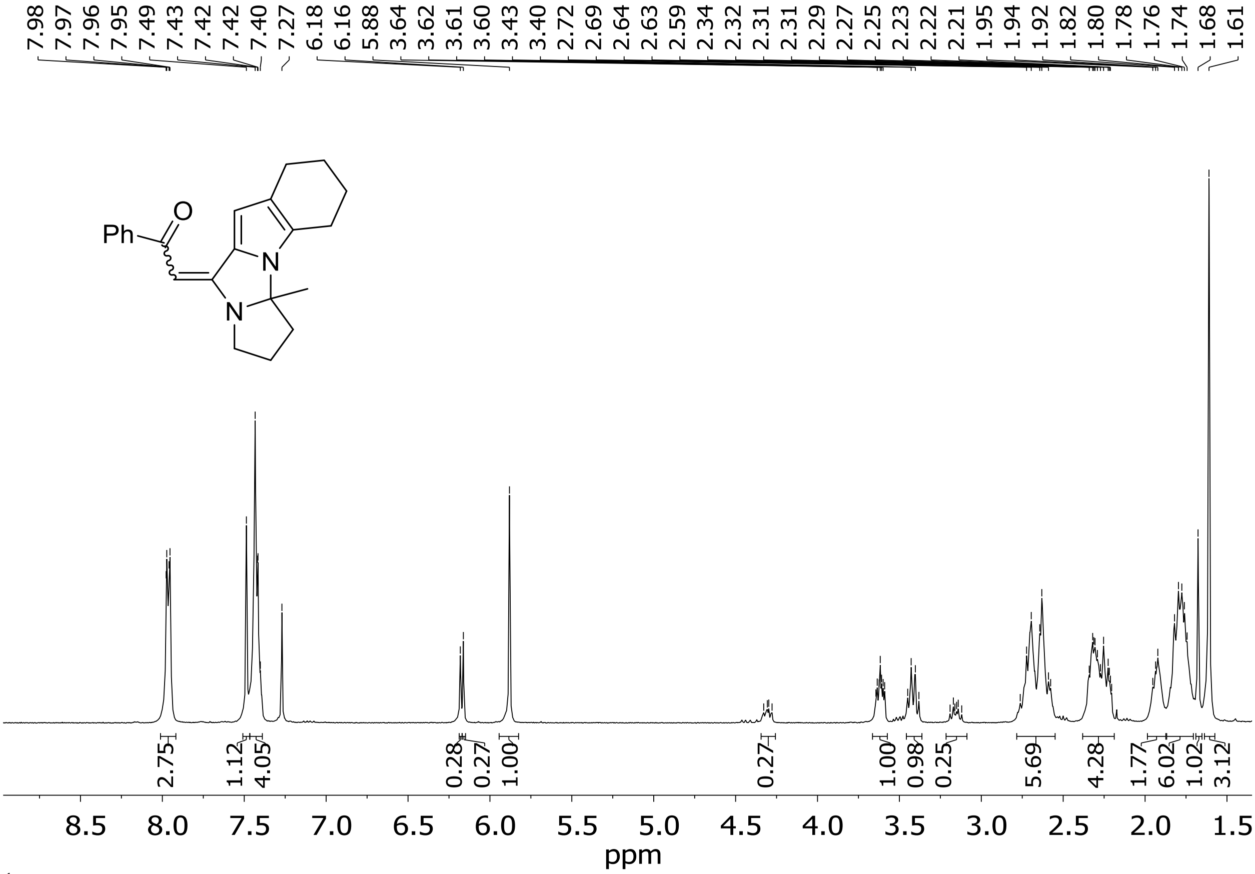

^{1}H NMR Spectrum of **5b** (400.1 MHz, $CDCl_3$)

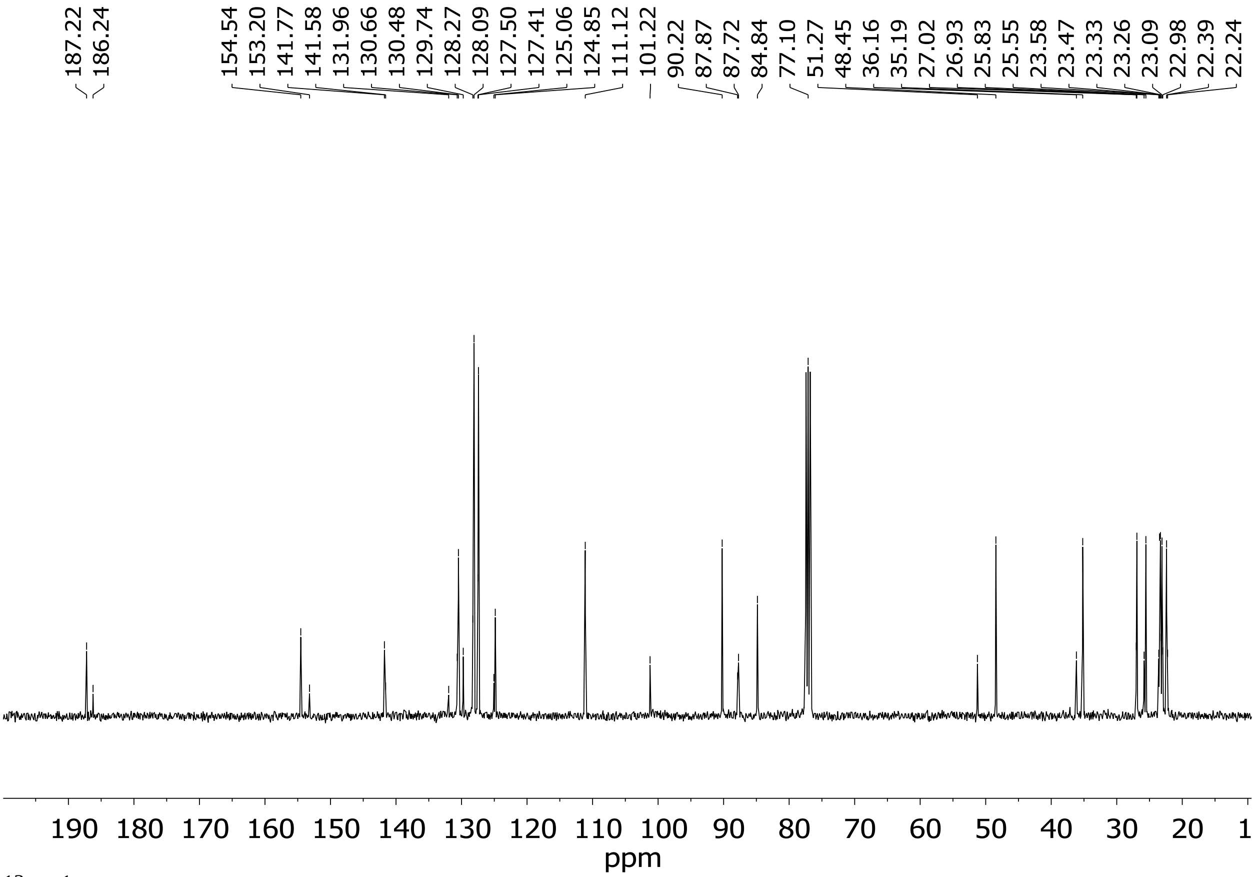

^{13}C{^{1}H} NMR Spectrum of **5b** (100.6 MHz, $CDCl_3$)

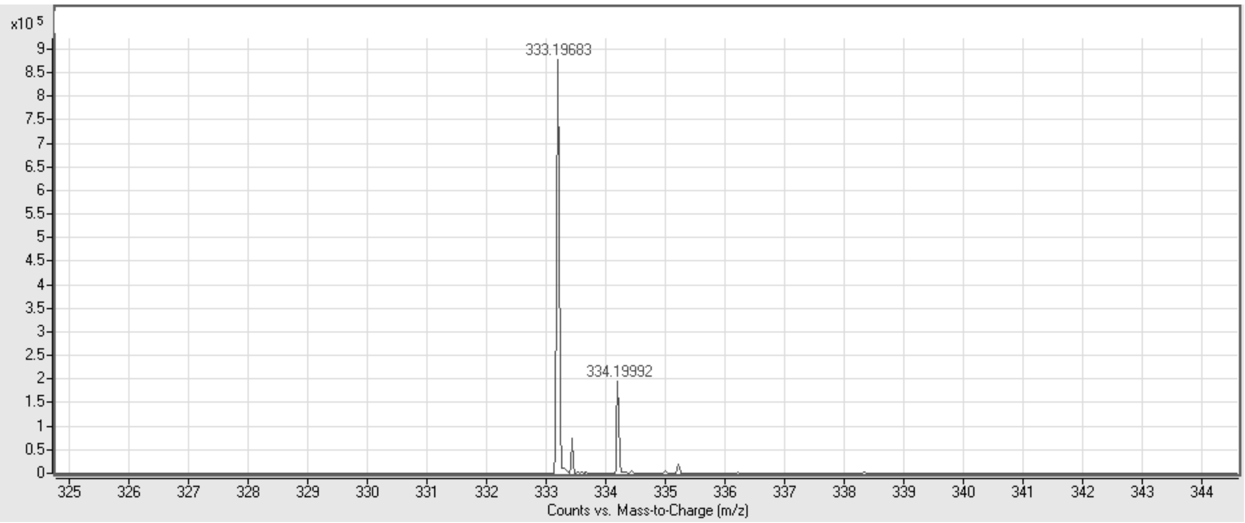

HRMS Spectrum of **5b**

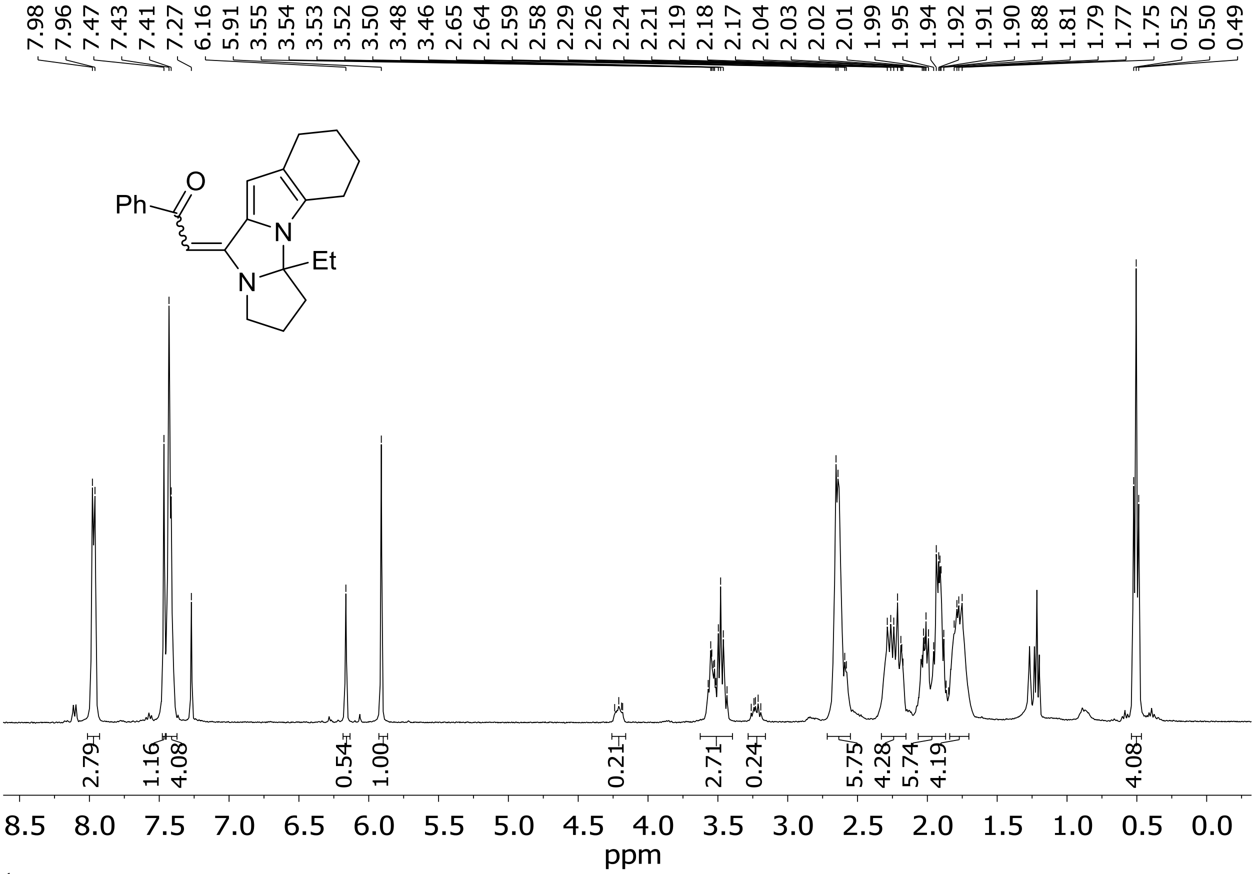

^{1}H NMR Spectrum of **5c** (400.1 MHz, $CDCl_3$)

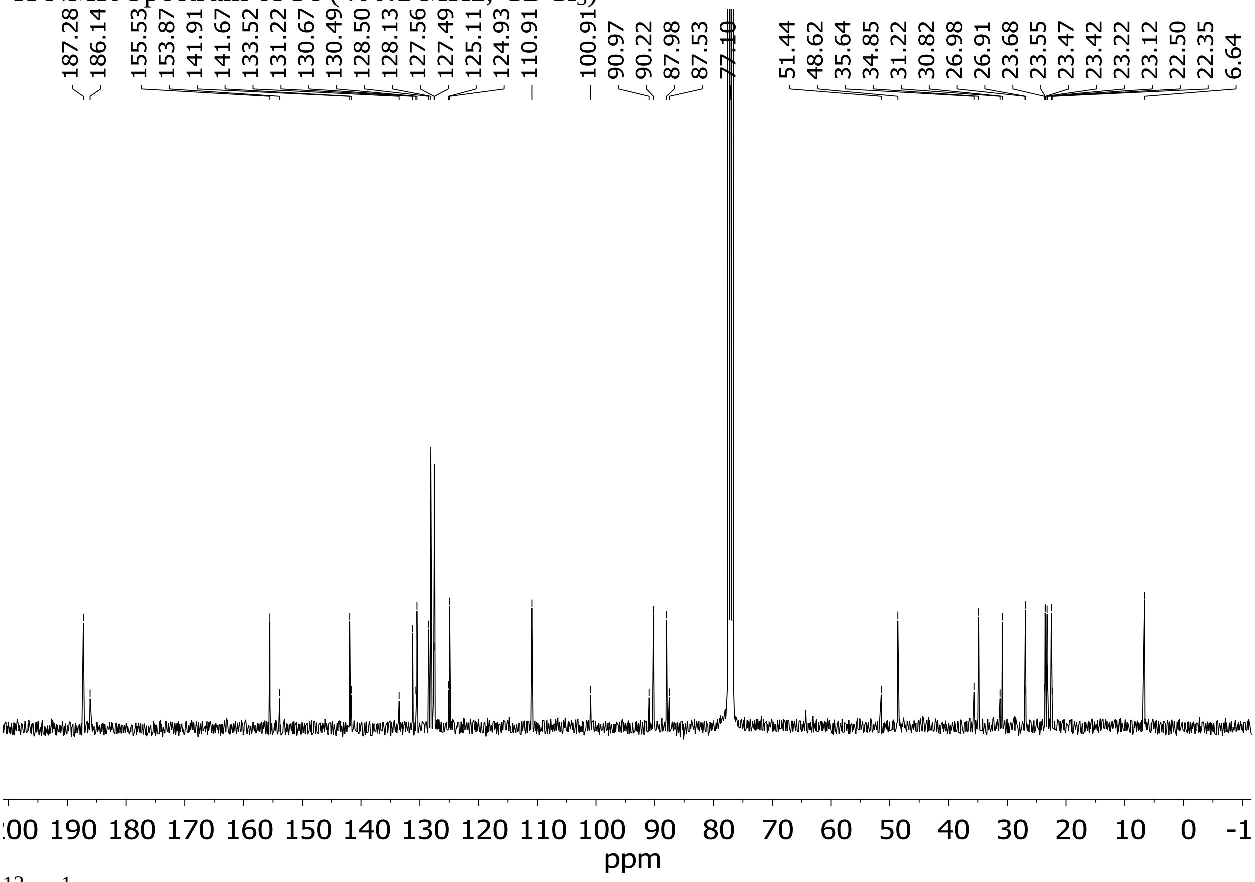

$^{13}C\{^{1}H\}$ NMR Spectrum of **5c** (100.6 MHz, $CDCl_3$)

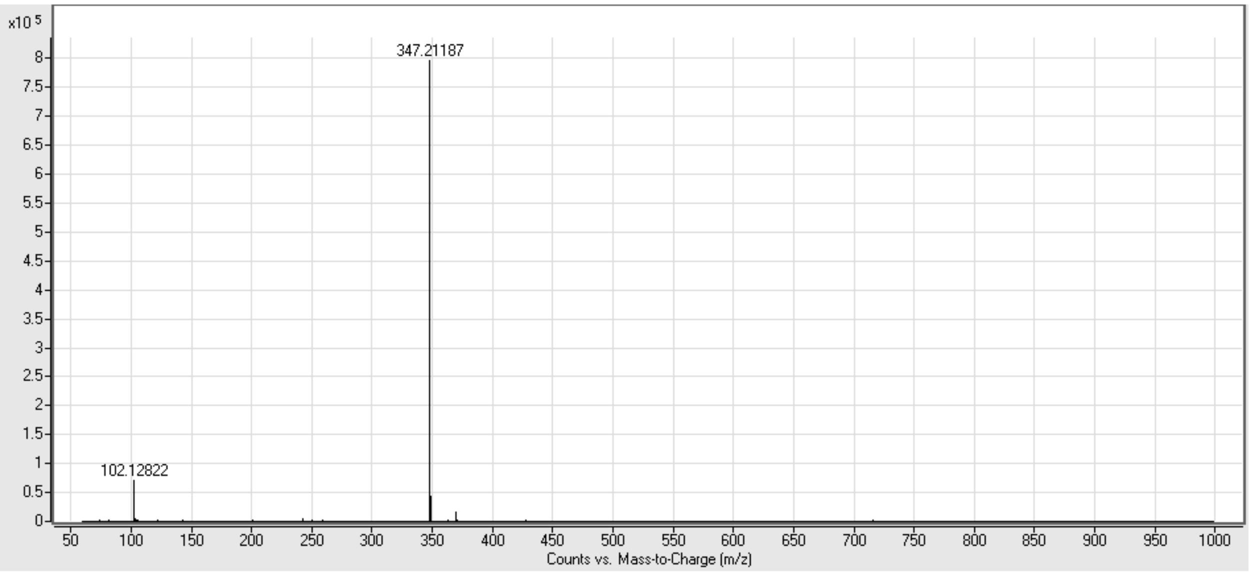

HRMS Spectrum of **5c**

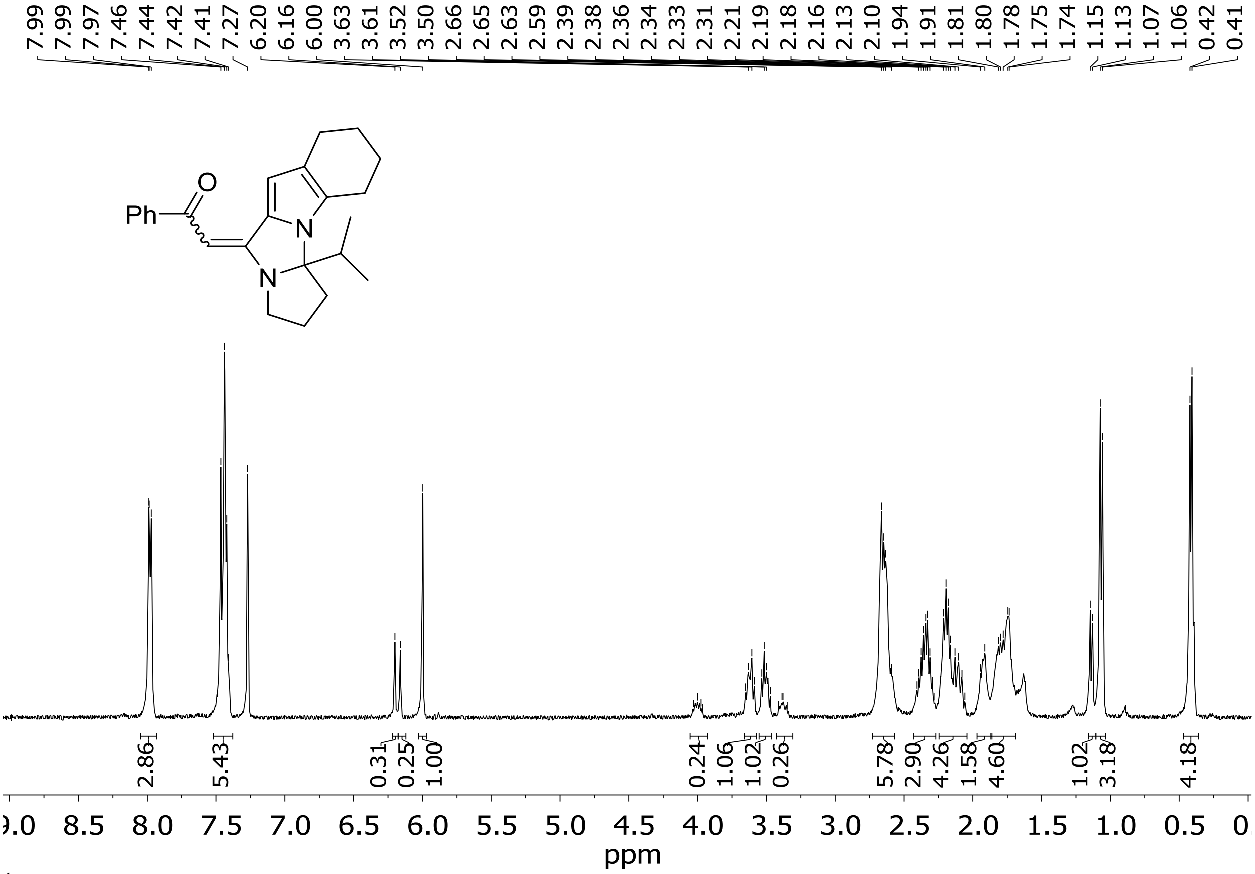

^{1}H NMR Spectrum of **5d** (400.1 MHz, $CDCl_3$)

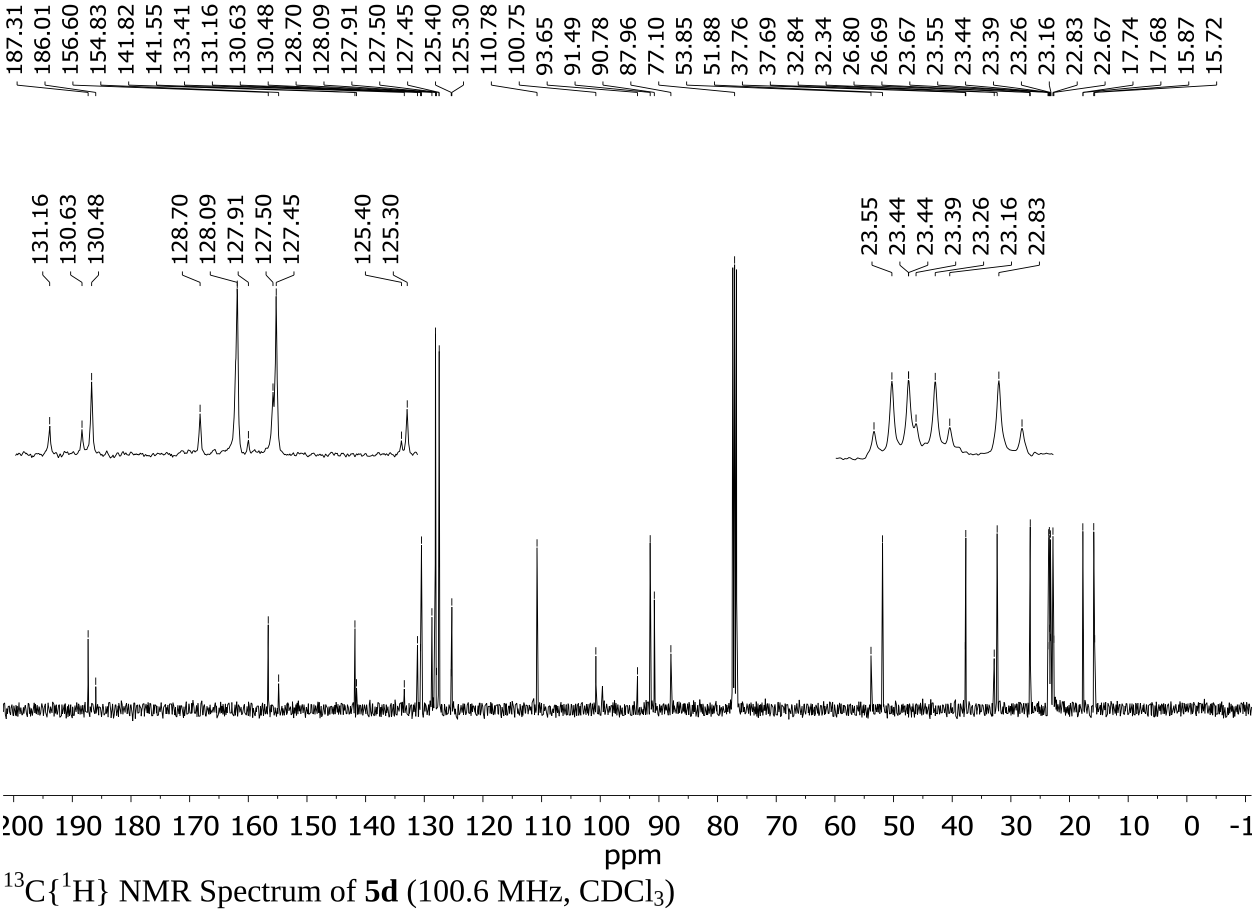

^{13}C{^{1}H} NMR Spectrum of **5d** (100.6 MHz, $CDCl_3$)

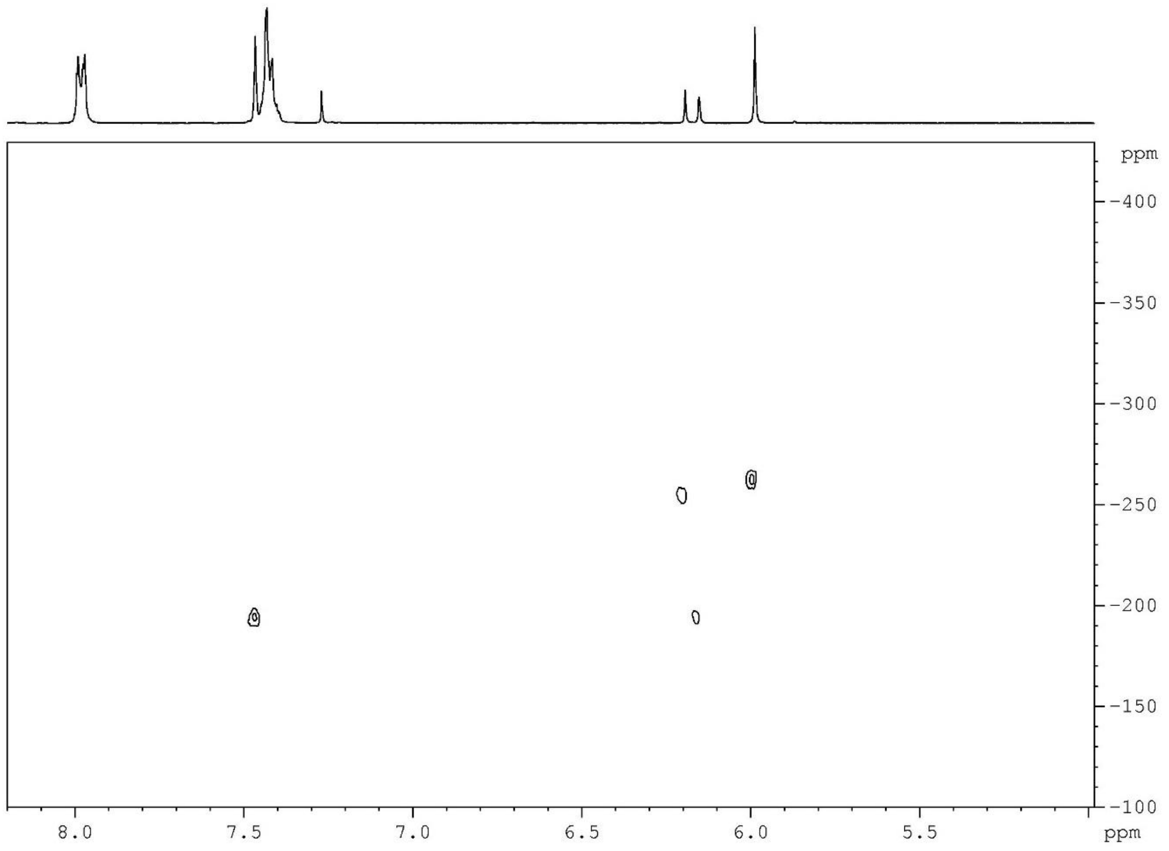

2D ^{1}H-^{15}N HMBC spectrum of **5d**

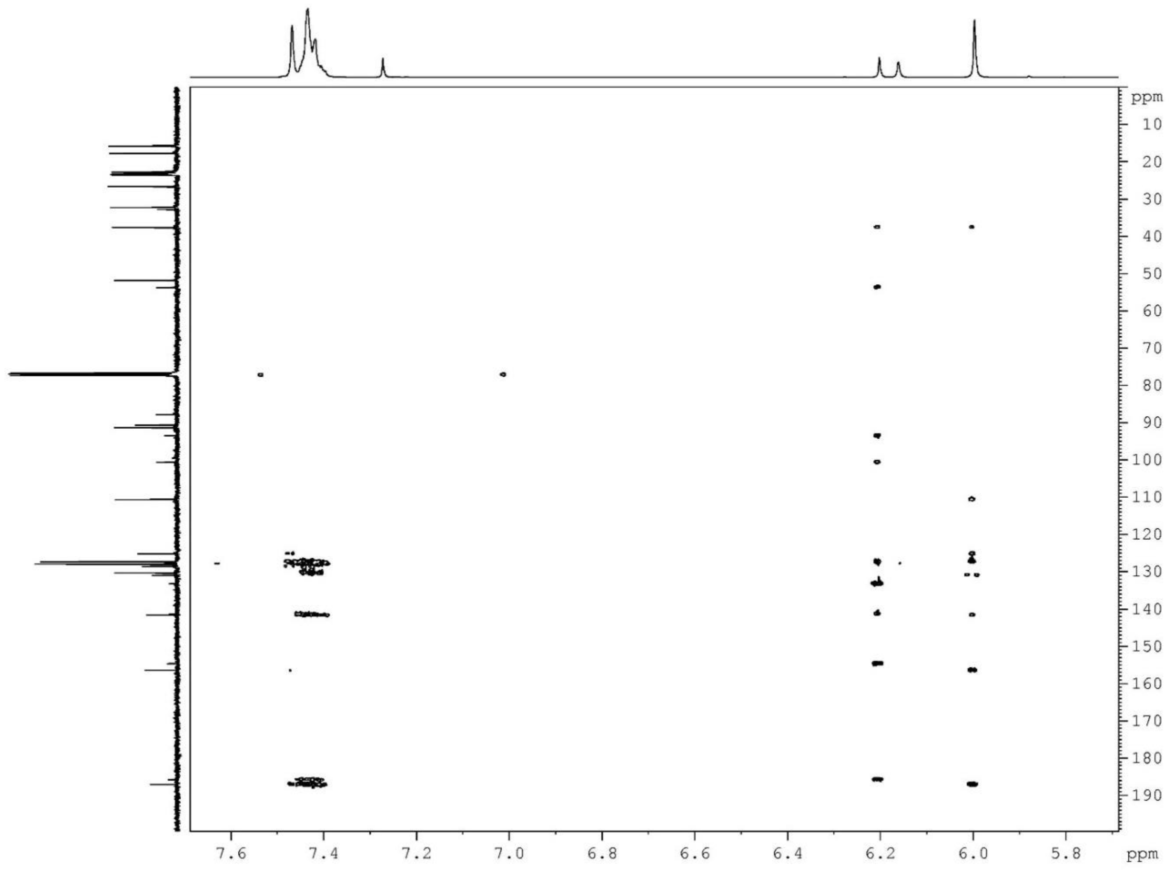

2D ^{1}H-^{13}C HMBC spectrum of **5d** (aromatic region)

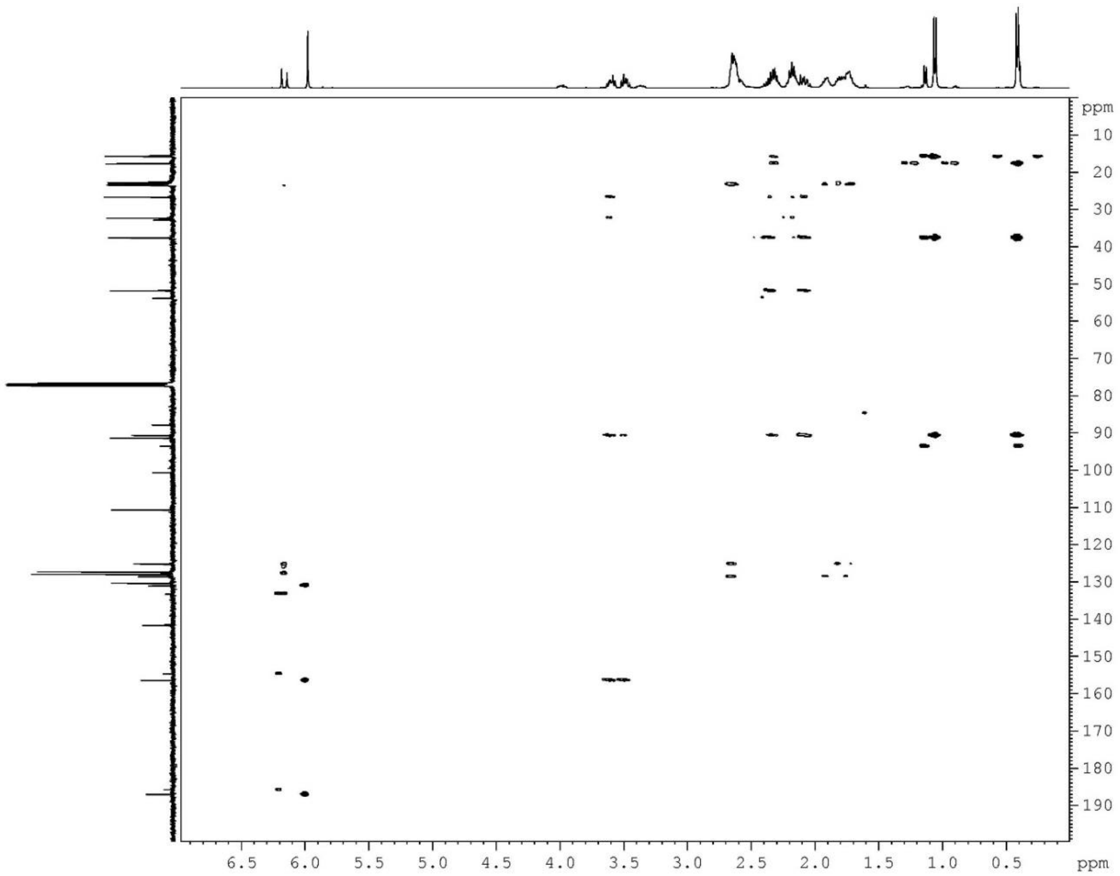

2D ^{1}H-^{13}C HMBC spectrum of **5d** (aliphatic region)

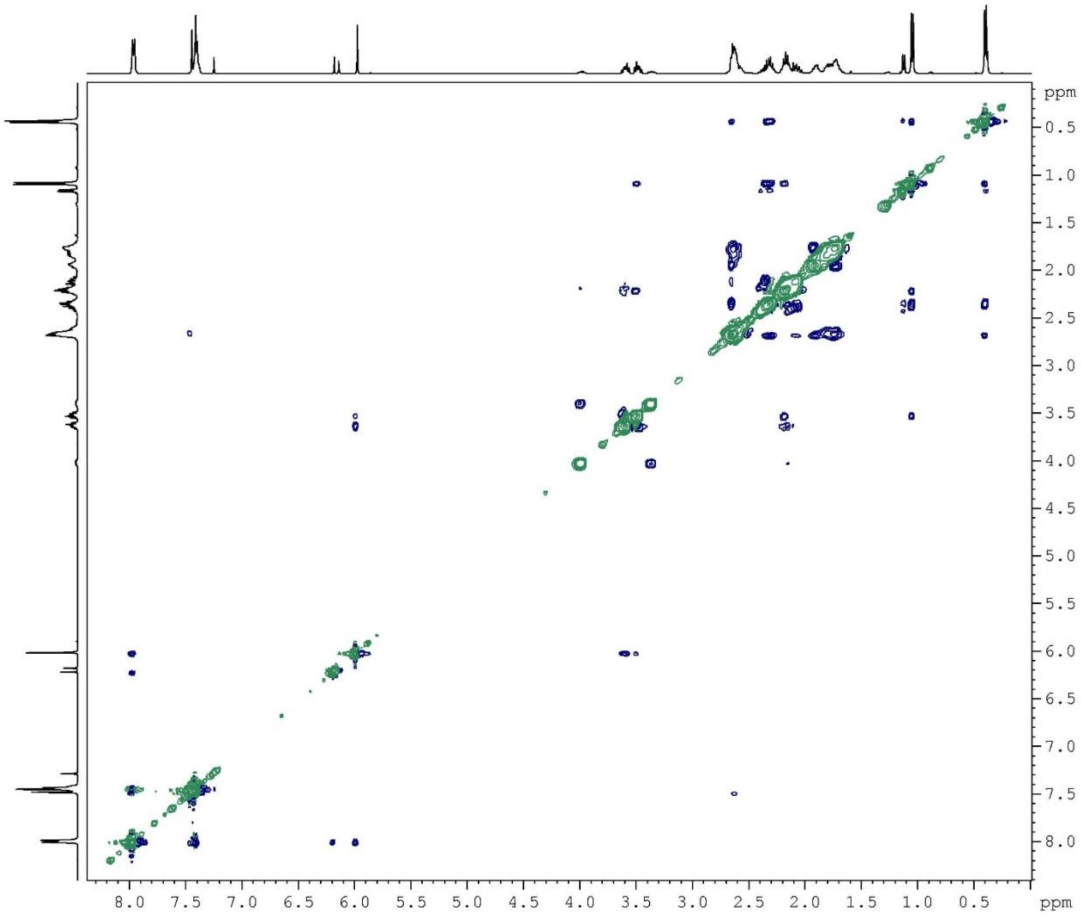

2D NOESY spectrum of **5d**

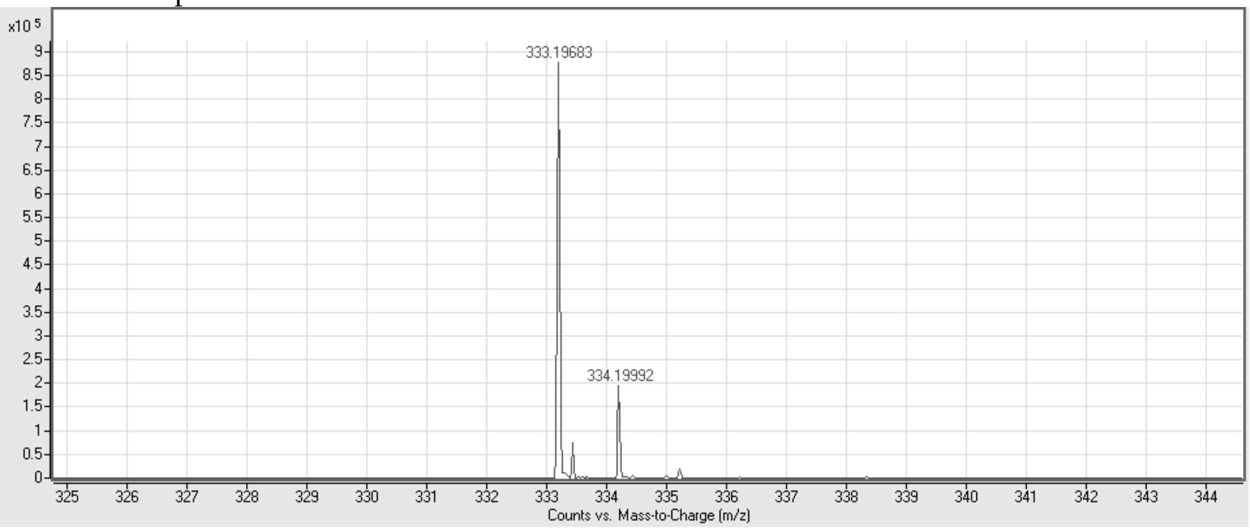

HRMS Spectrum of **5d**

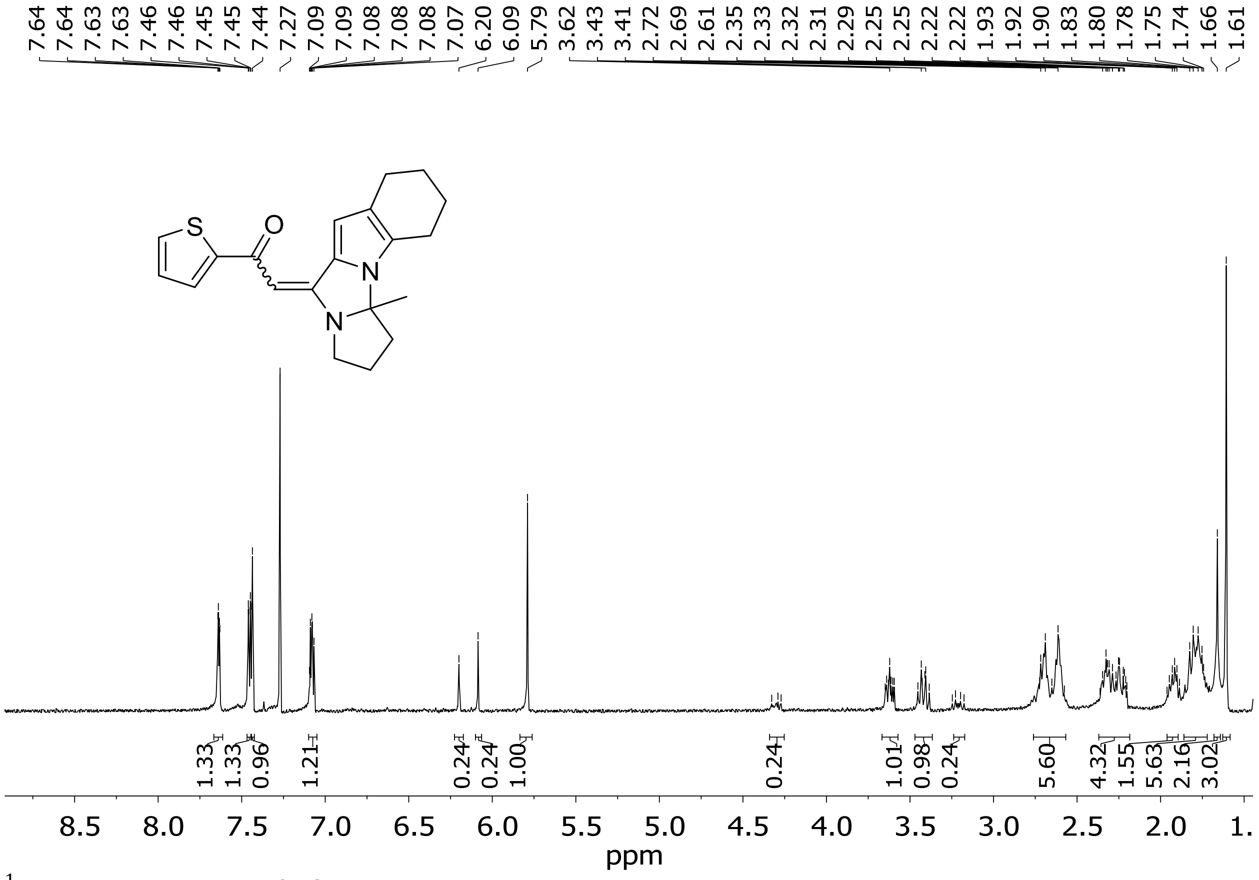

^{1}H NMR Spectrum of **5f** (400.1 MHz, $CDCl_3$)

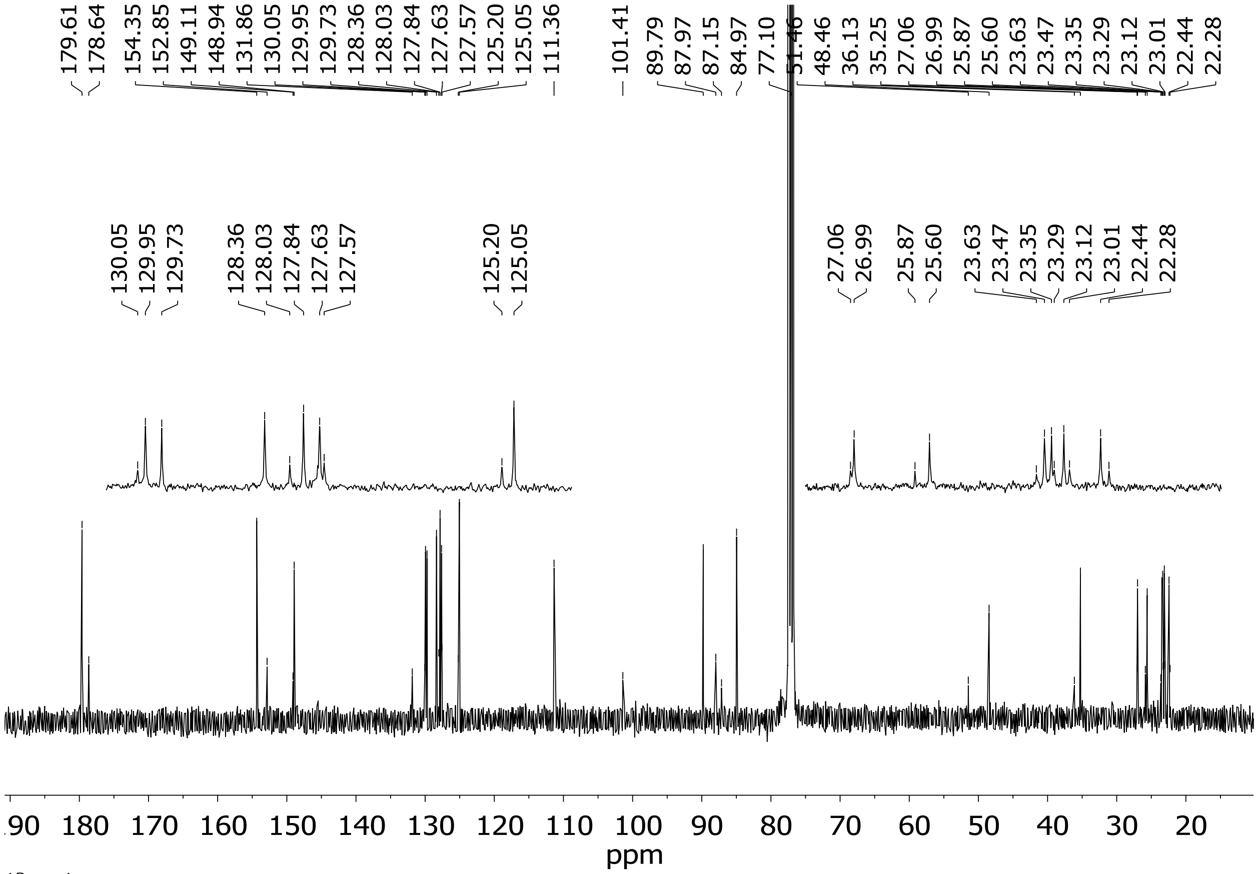

$^{13}C\{^{1}H\}$ NMR Spectrum of **5f** (100.6 MHz, $CDCl_3$)

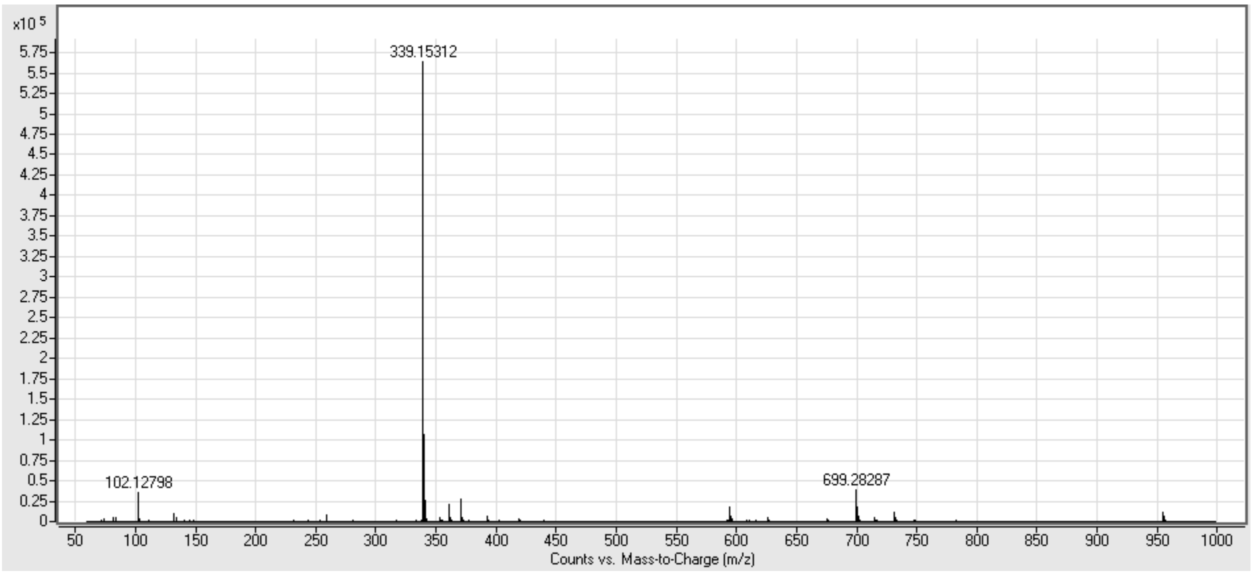

HRMS Spectrum of **5f**

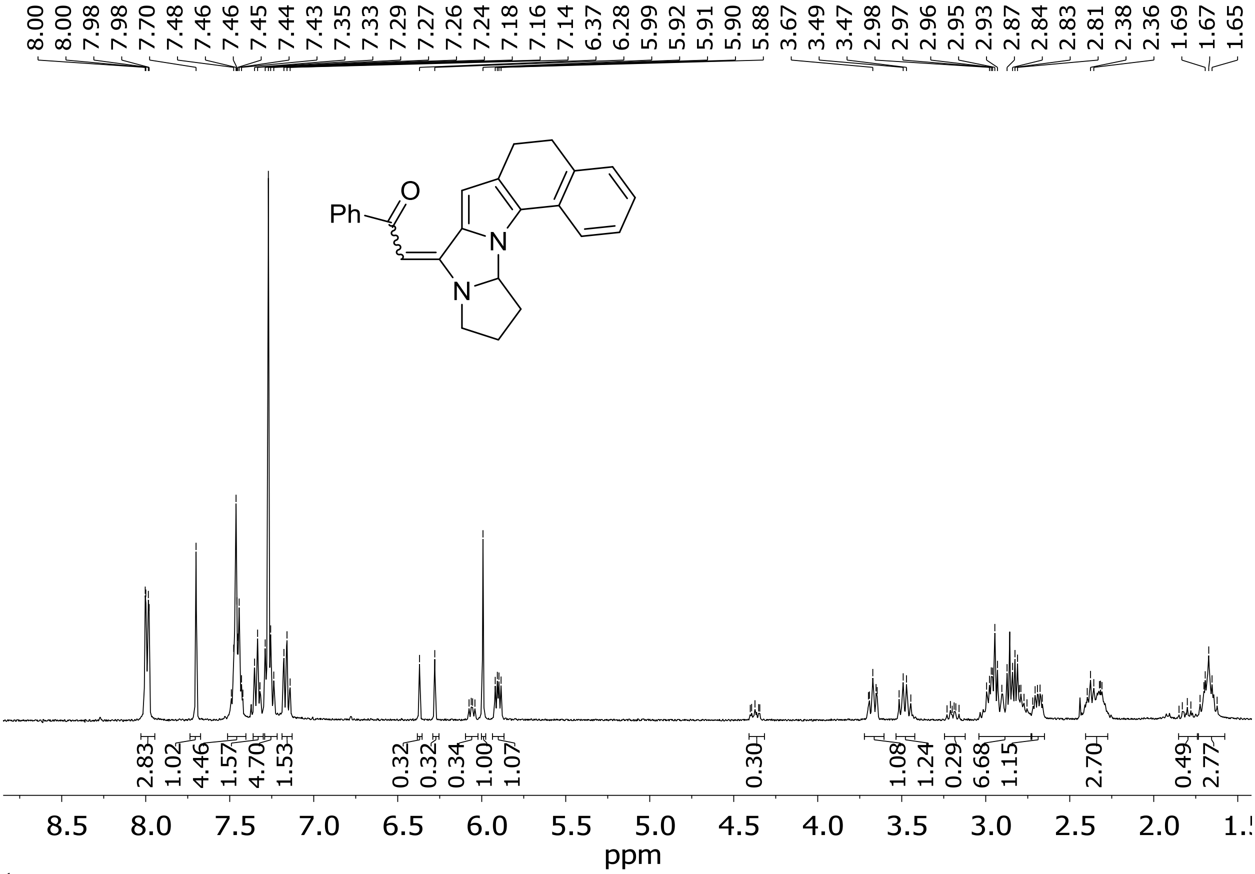

^{1}H NMR Spectrum of **6a** (400.1 MHz, $CDCl_3$)

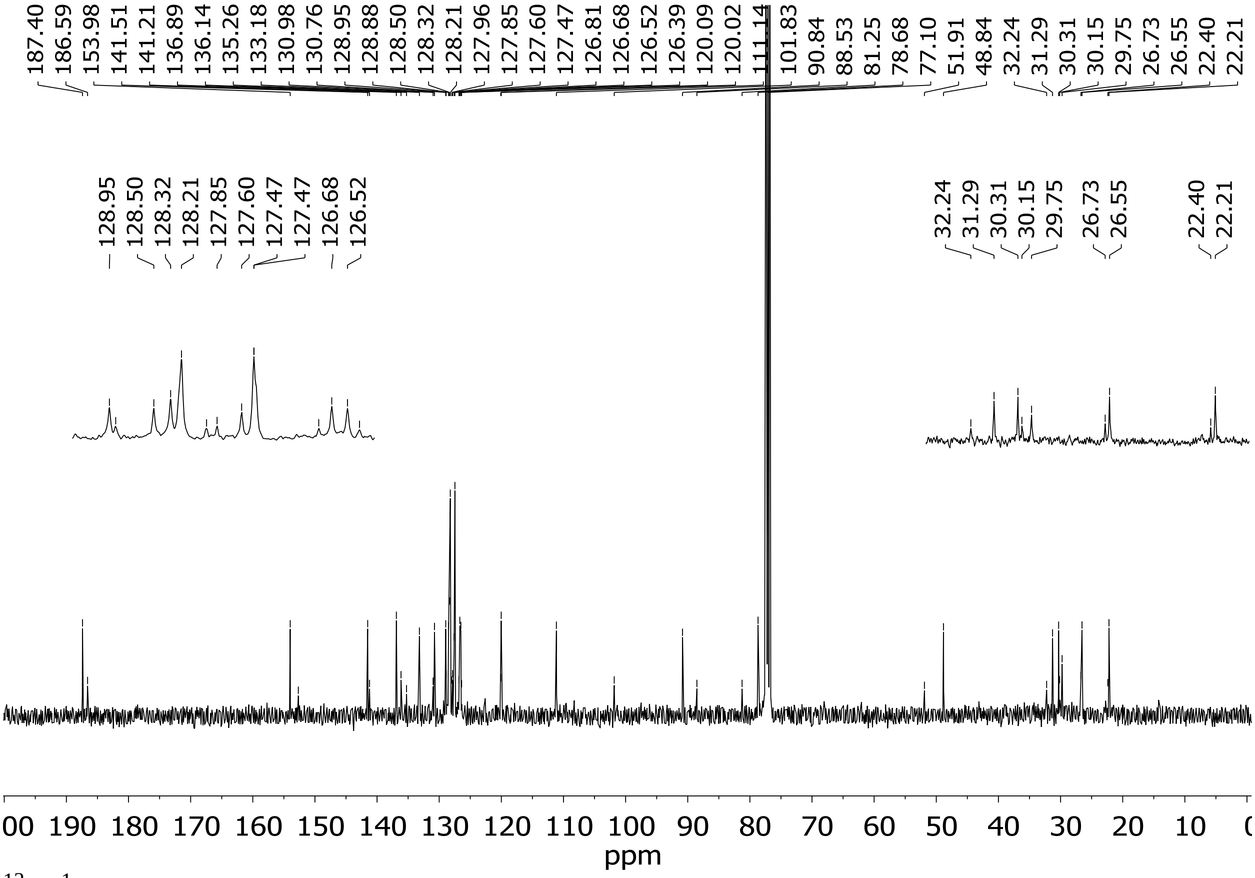

^{13}C{^{1}H} NMR Spectrum of **6a** (100.6 MHz, $CDCl_3$)

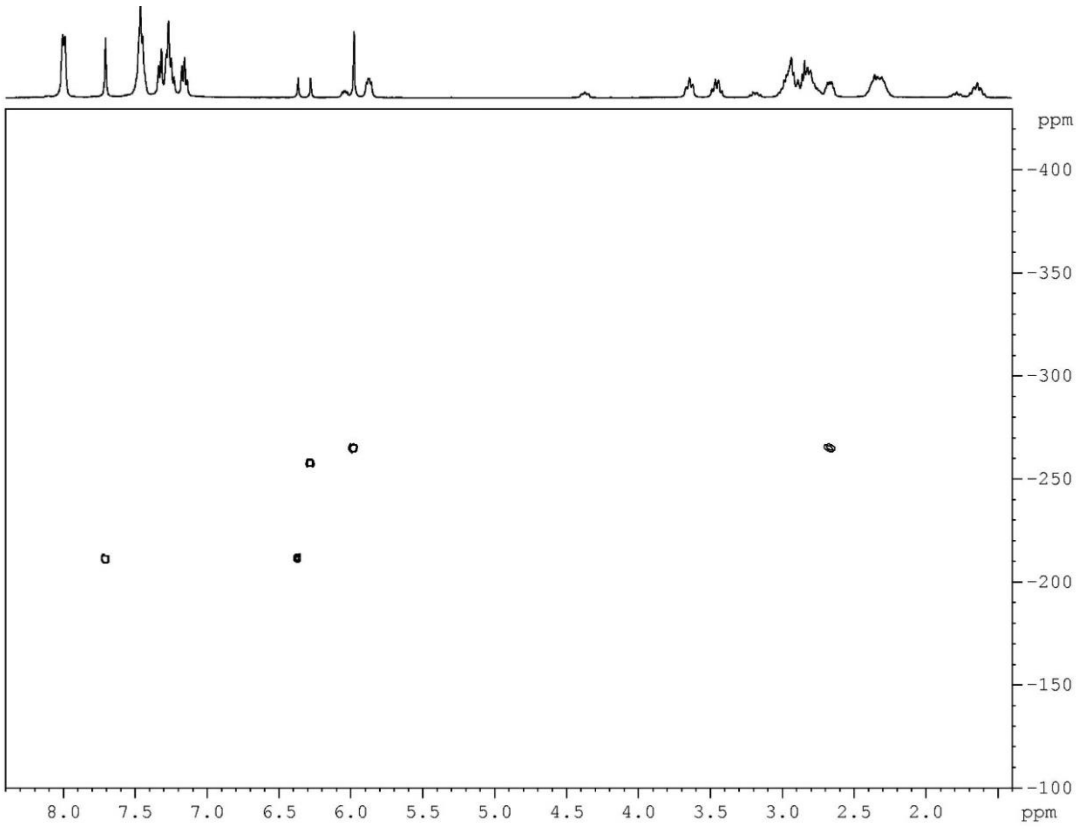

2D ^{1}H-^{15}N HMBC spectrum of **6a**

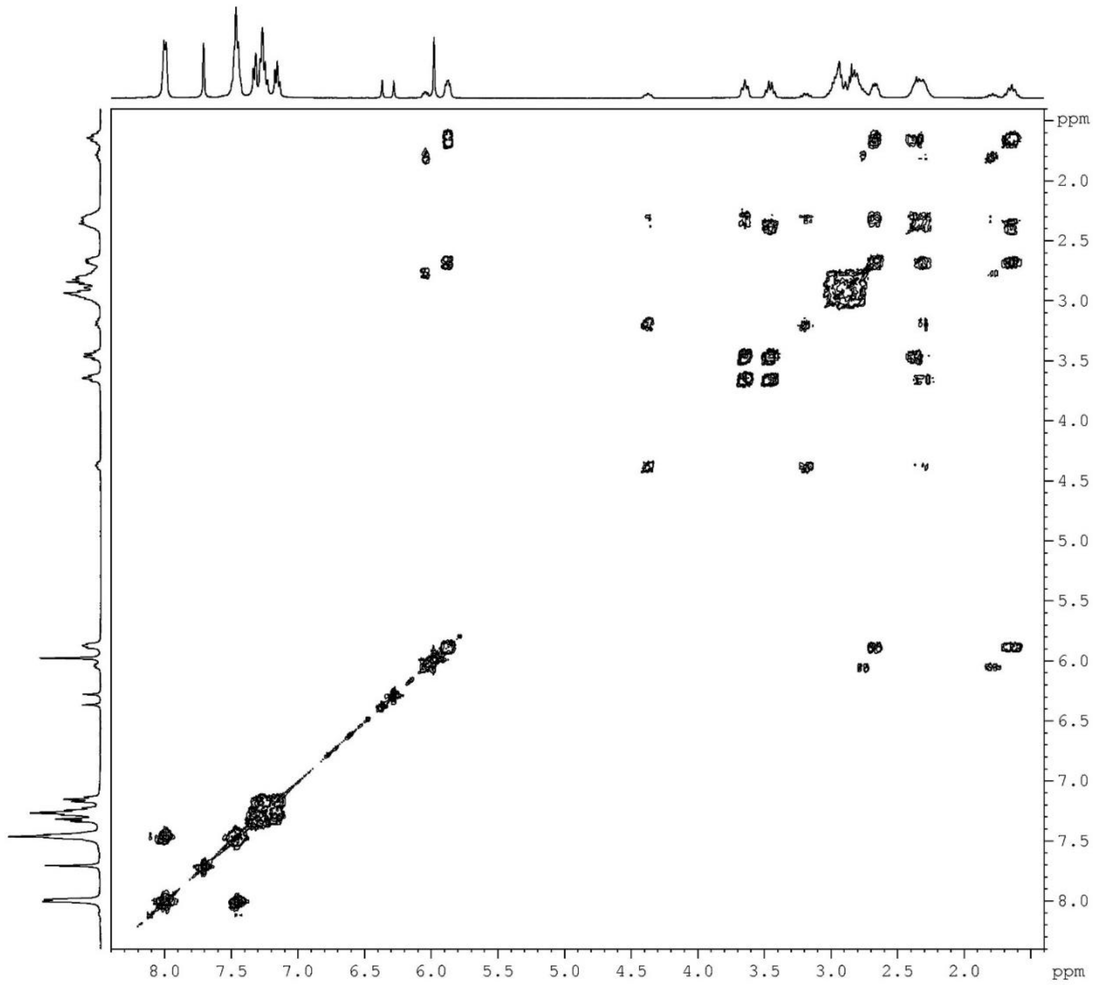

2D COSY spectrum of **6a**

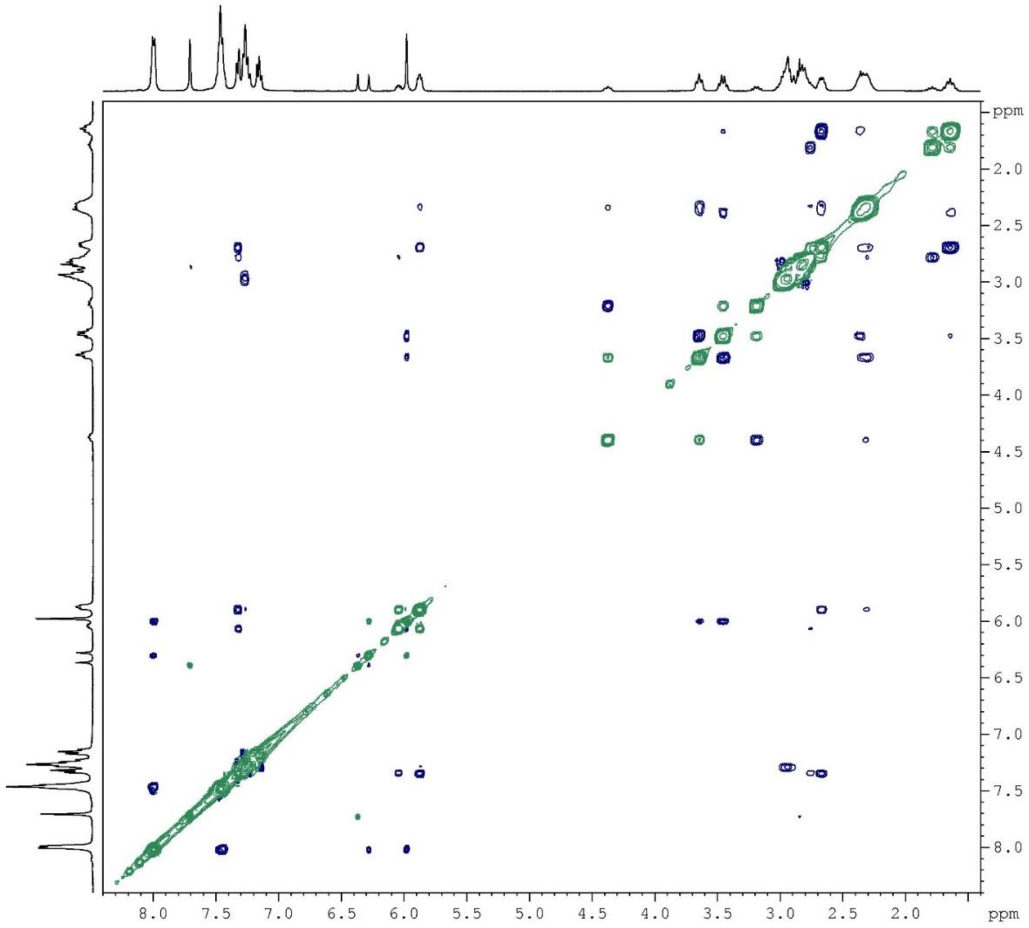

2D NOESY spectrum of **6a**

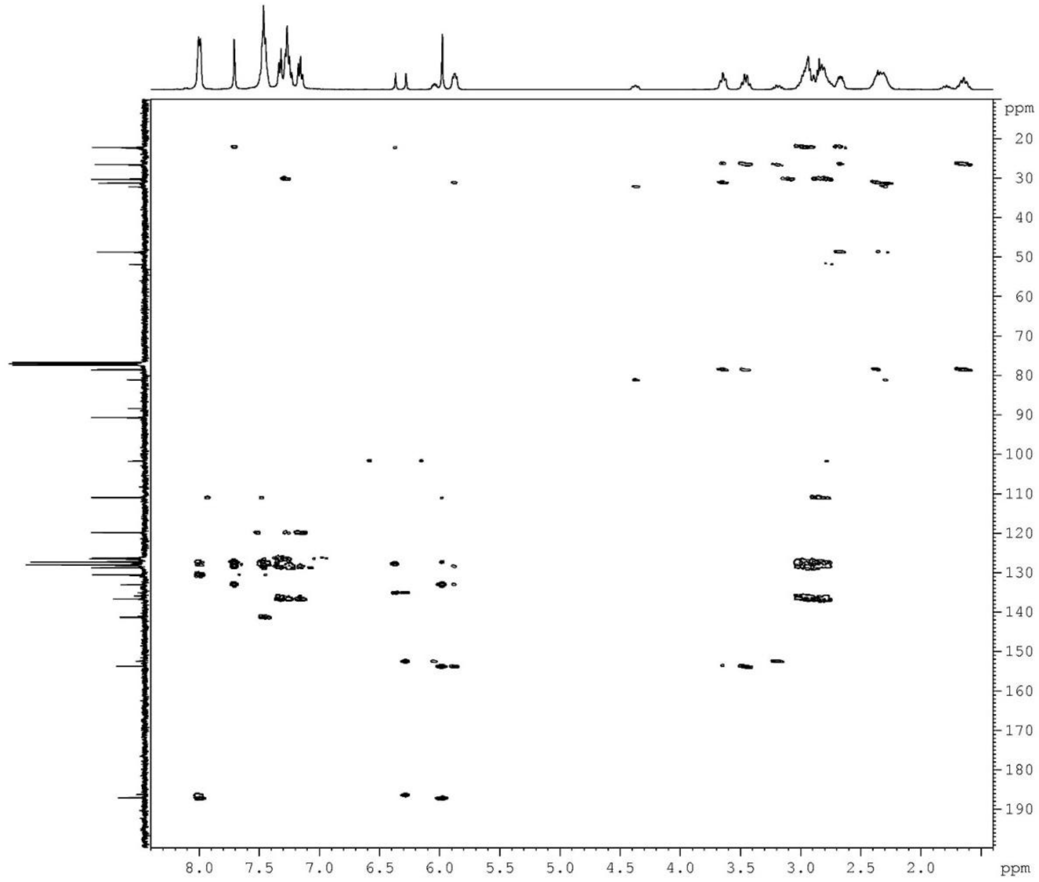

2D ^{1}H-^{13}C HMBC spectrum of **6a**

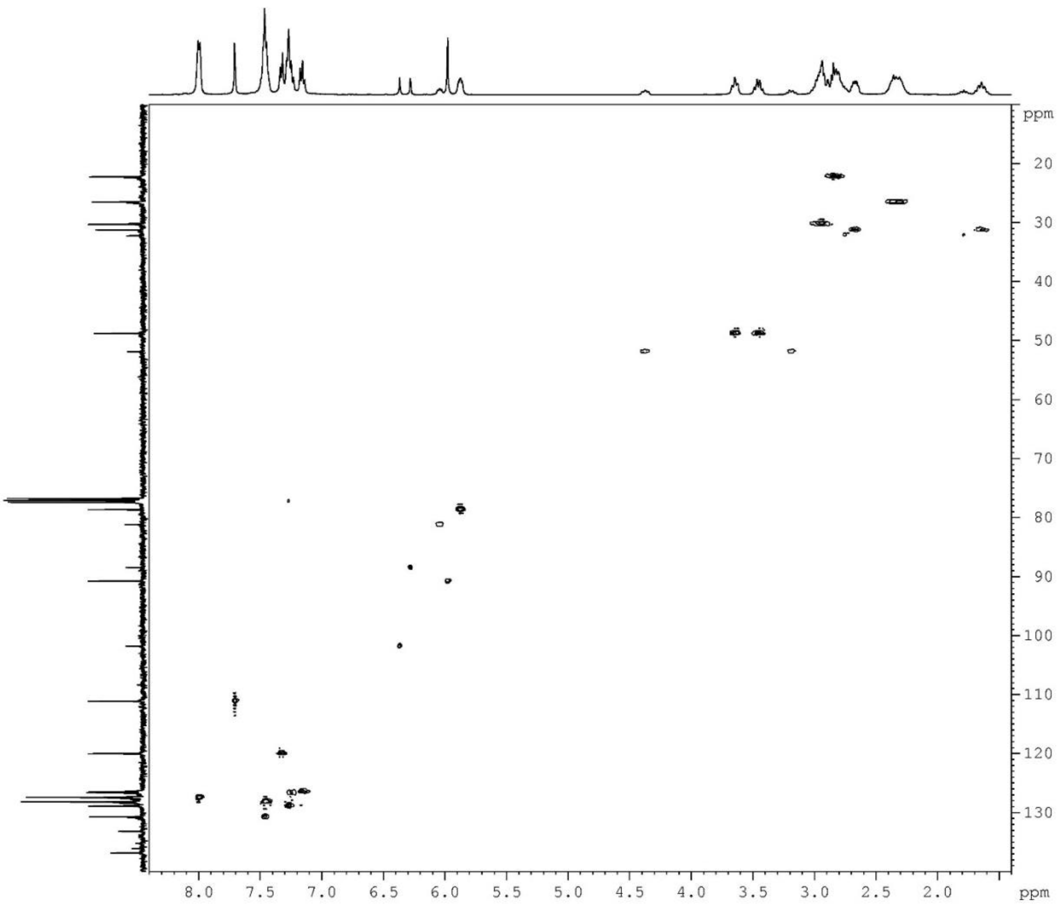

2D ^{1}H-^{13}C HSQC spectrum of **6a**

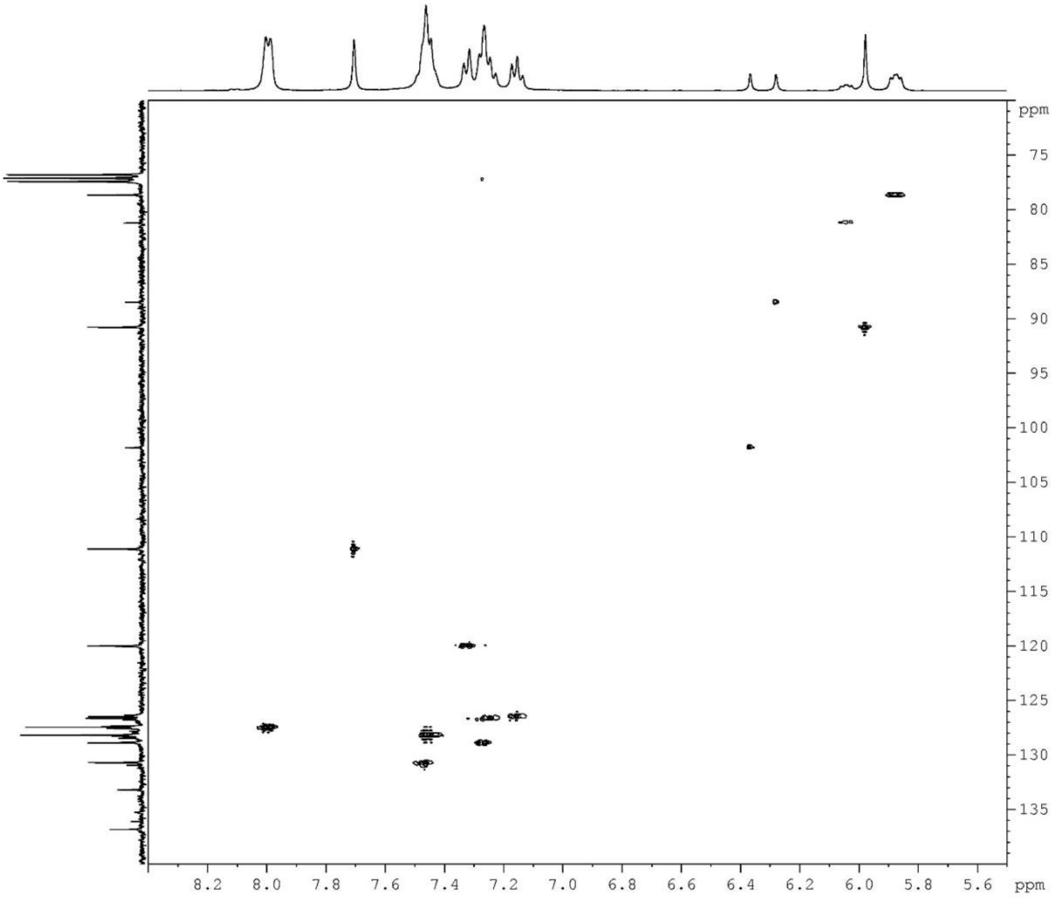

2D ^{1}H-^{13}C HSQC spectrum of **6a** (aromatic region)

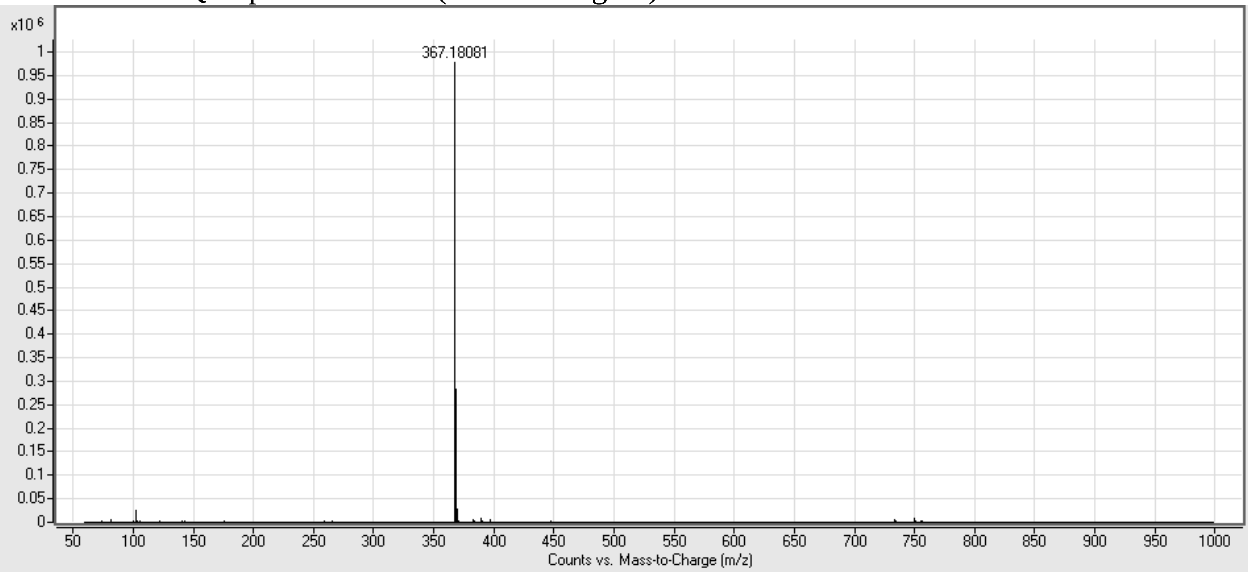

HRMS Spectrum of **6a**

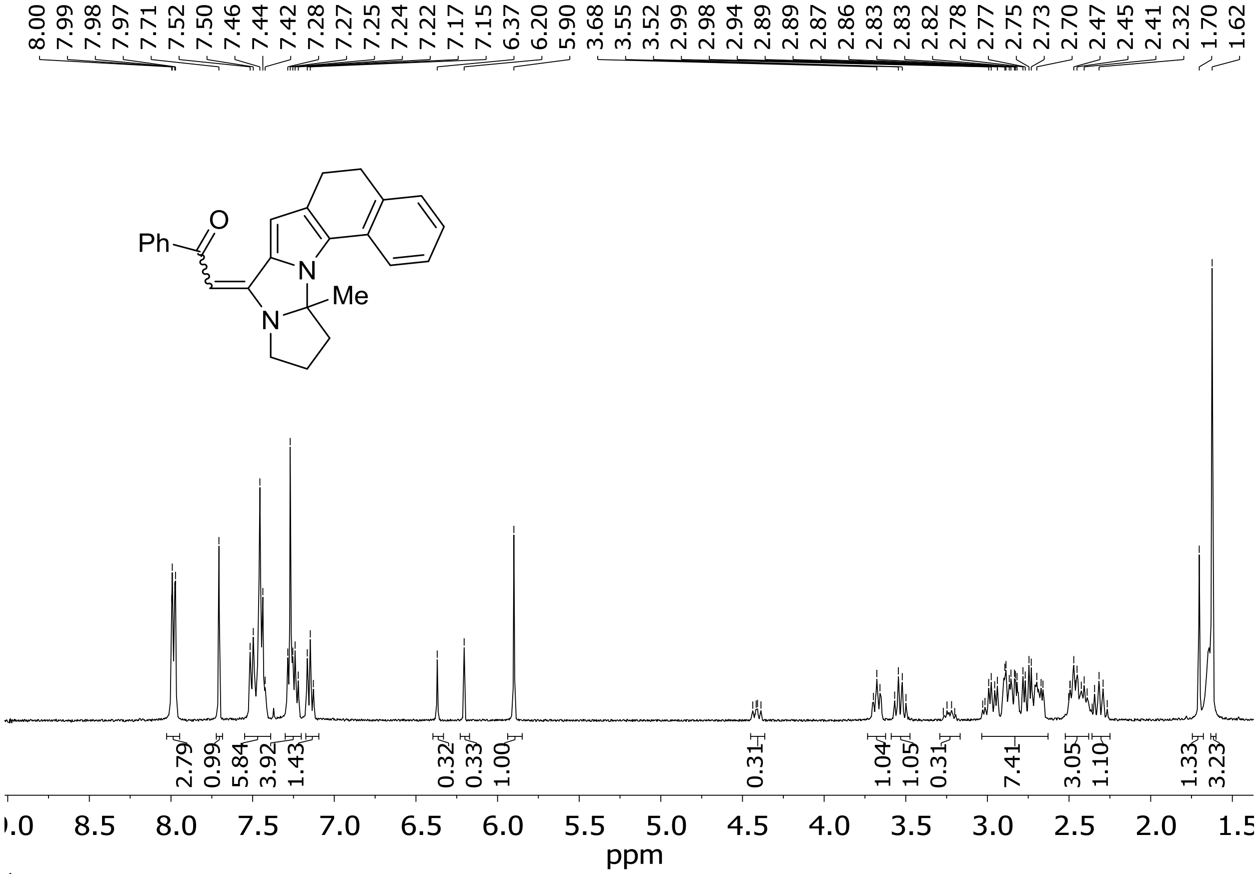

^{1}H NMR Spectrum of **6b** (400.1 MHz, $CDCl_3$)

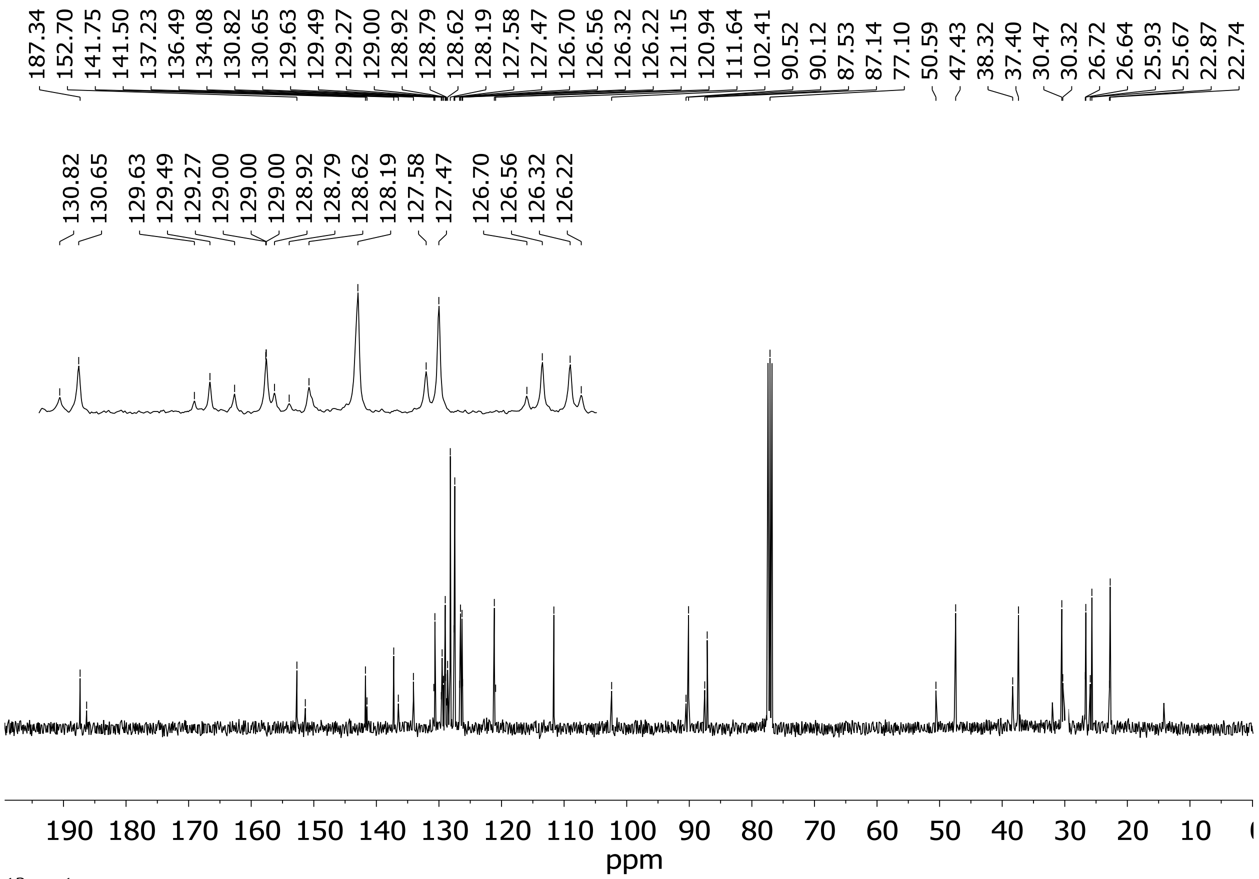

$^{13}C\{^1H\}$ NMR Spectrum of **6b** (100.6 MHz, $CDCl_3$)

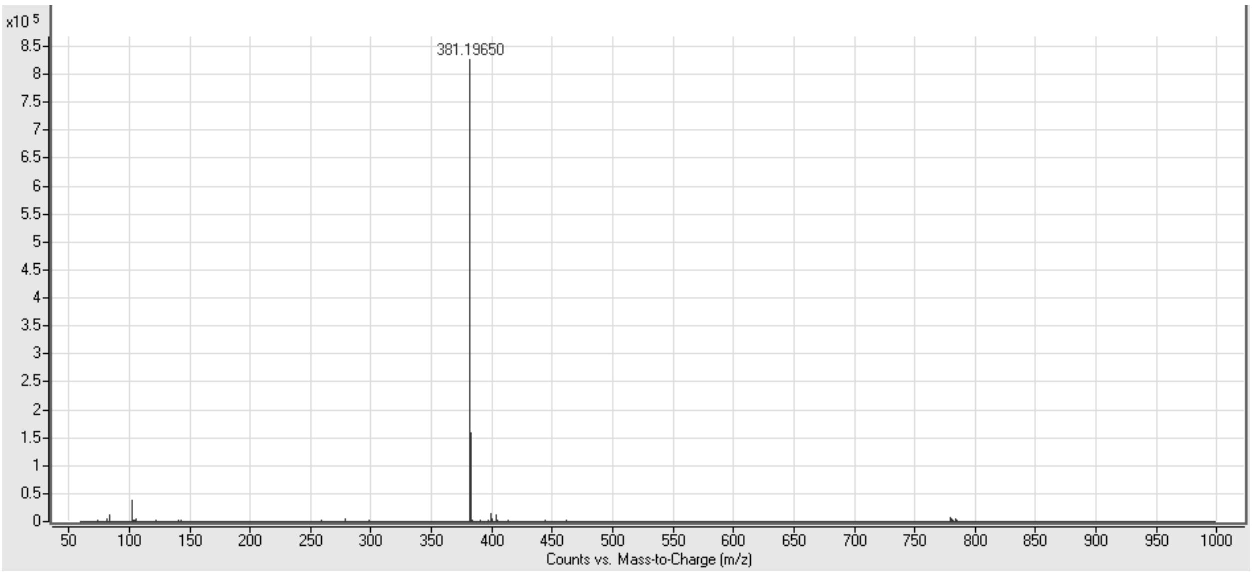

HRMS Spectrum of **6b**

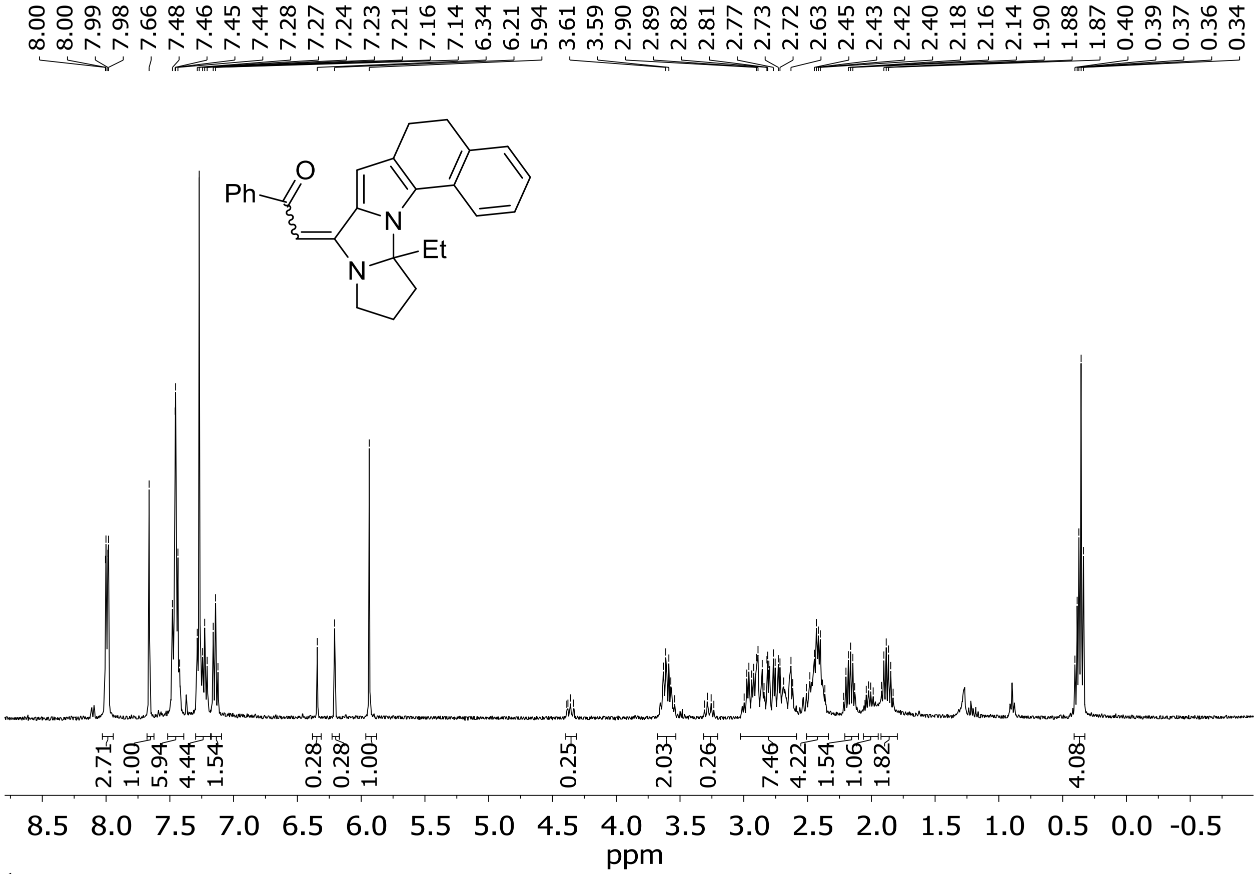

^{1}H NMR Spectrum of **6c** (400.1 MHz, $CDCl_3$)

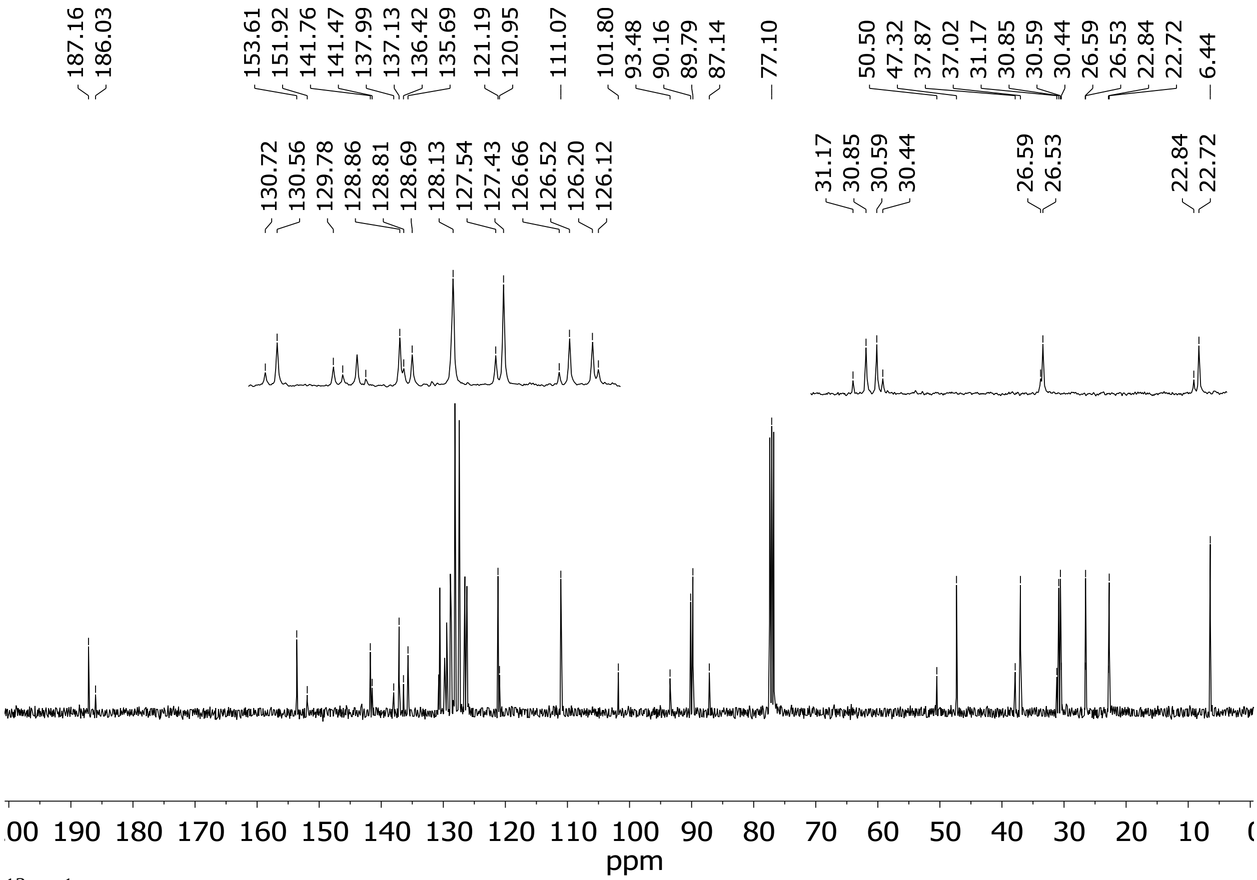

^{13}C{^{1}H} NMR Spectrum of **6c** (100.6 MHz, $CDCl_3$)

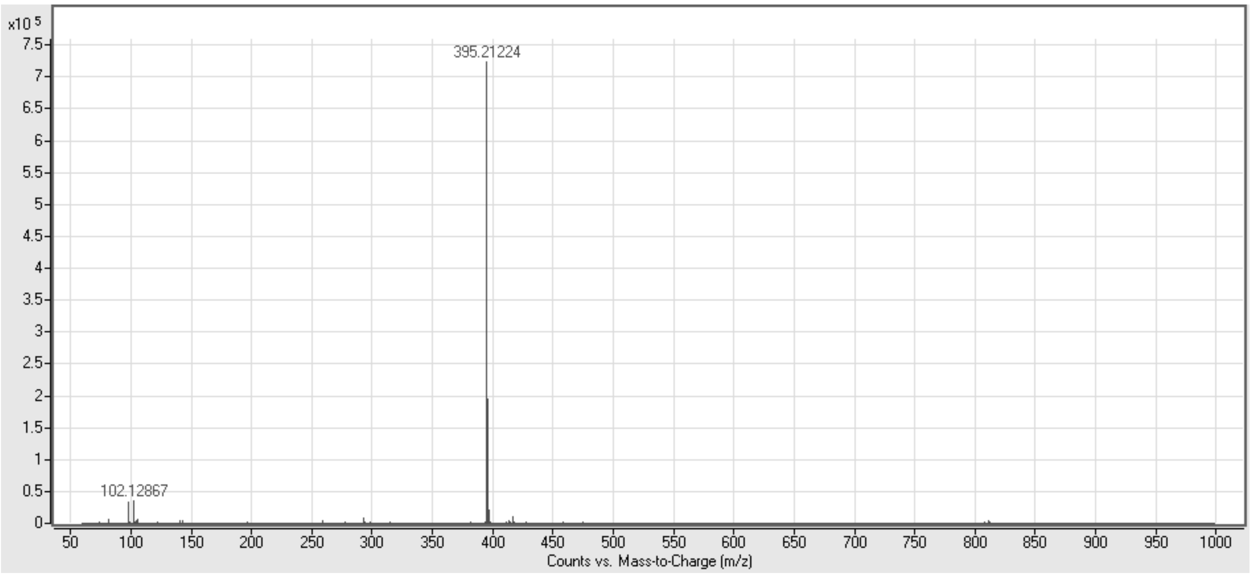

HRMS Spectrum of **6c**

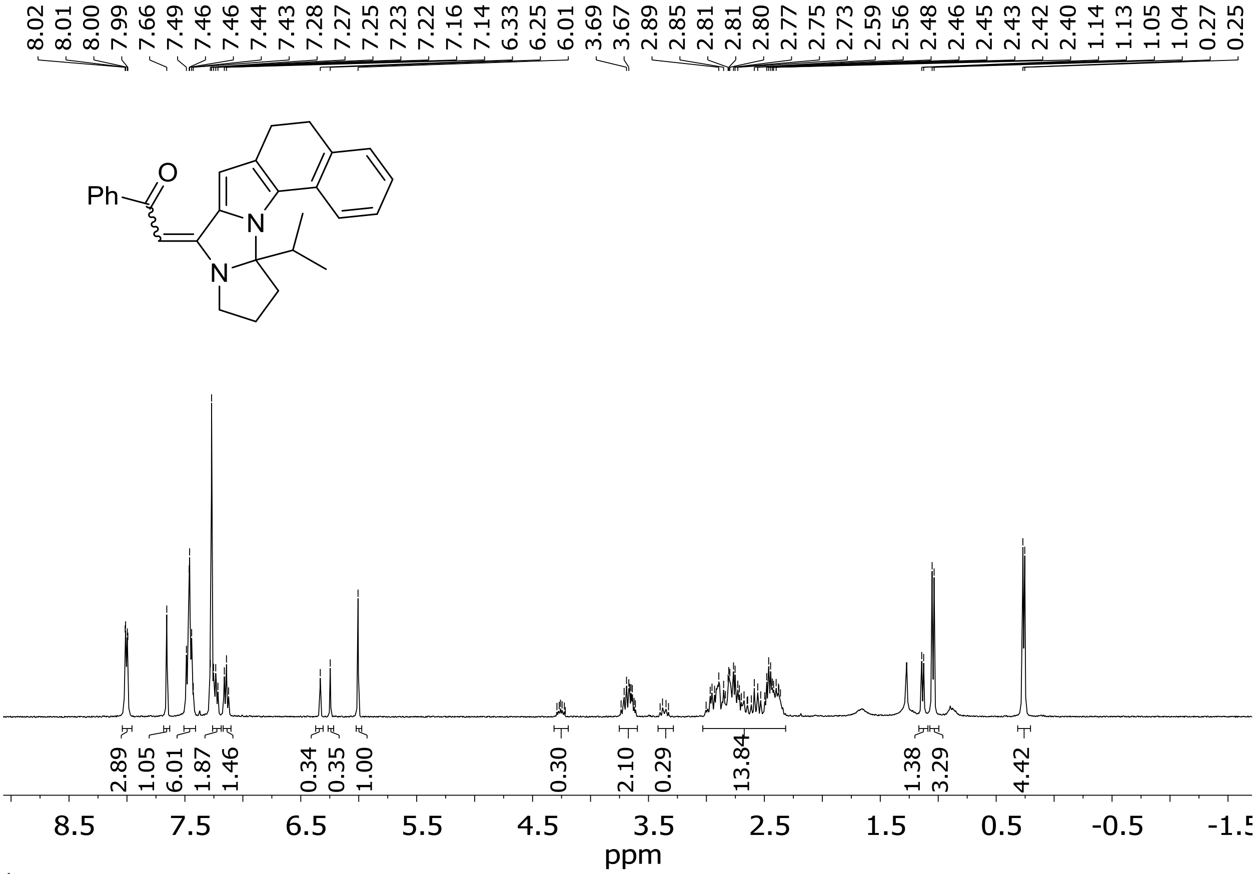

^{1}H NMR Spectrum of **6d** (400.1 MHz, $CDCl_3$)

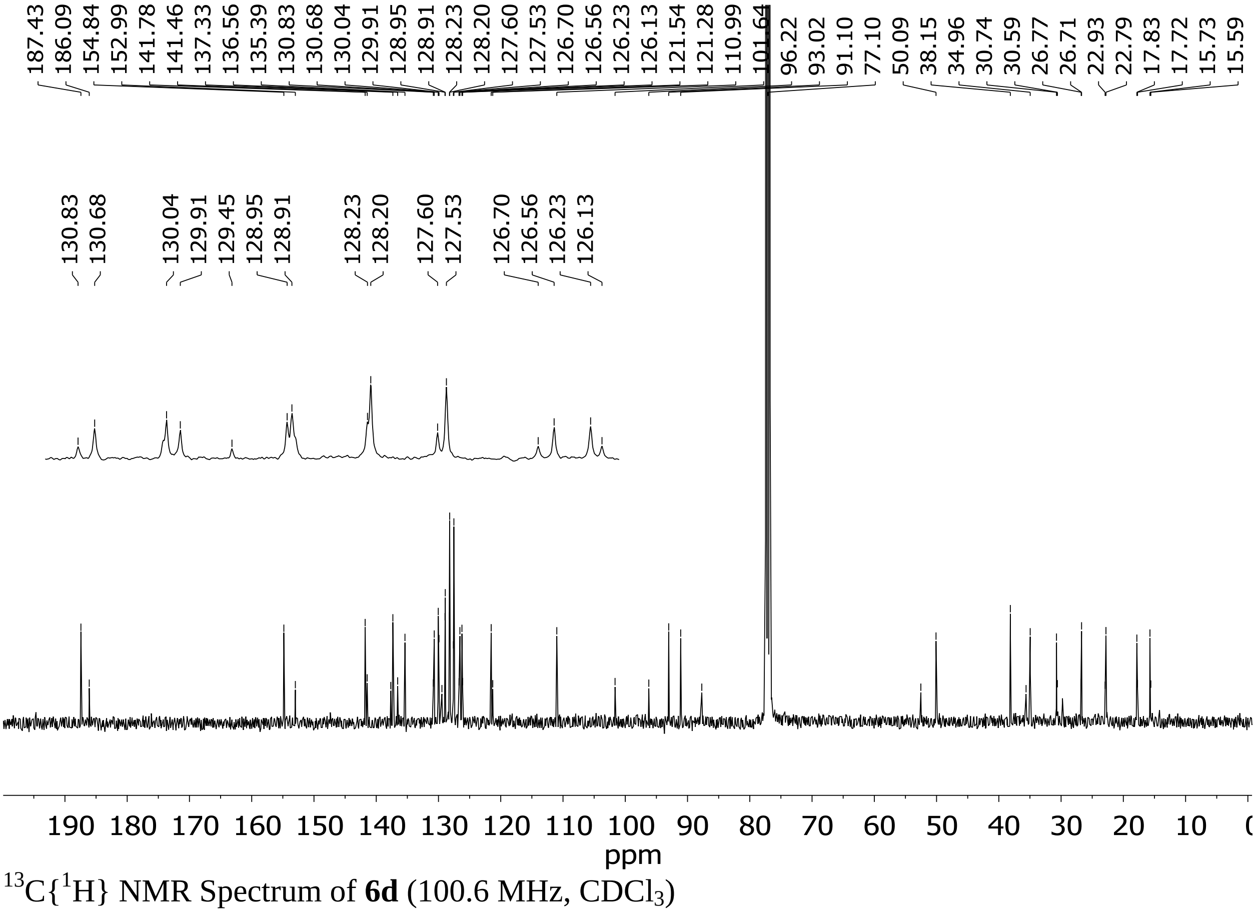

^{13}C{^{1}H} NMR Spectrum of **6d** (100.6 MHz, $CDCl_3$)

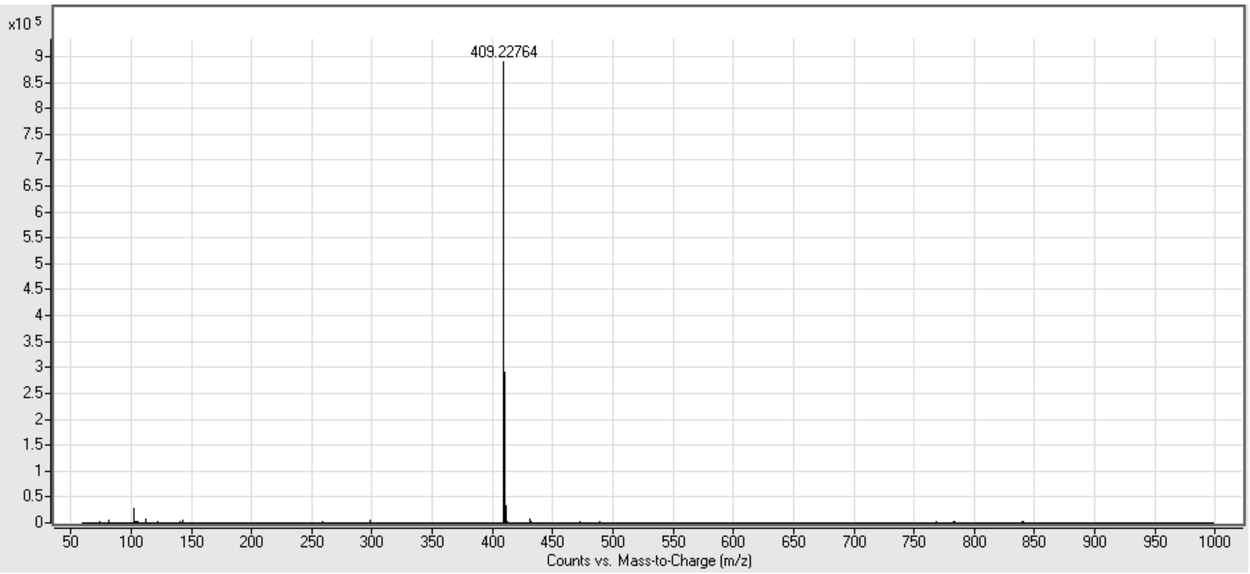

HRMS Spectrum of **6d**

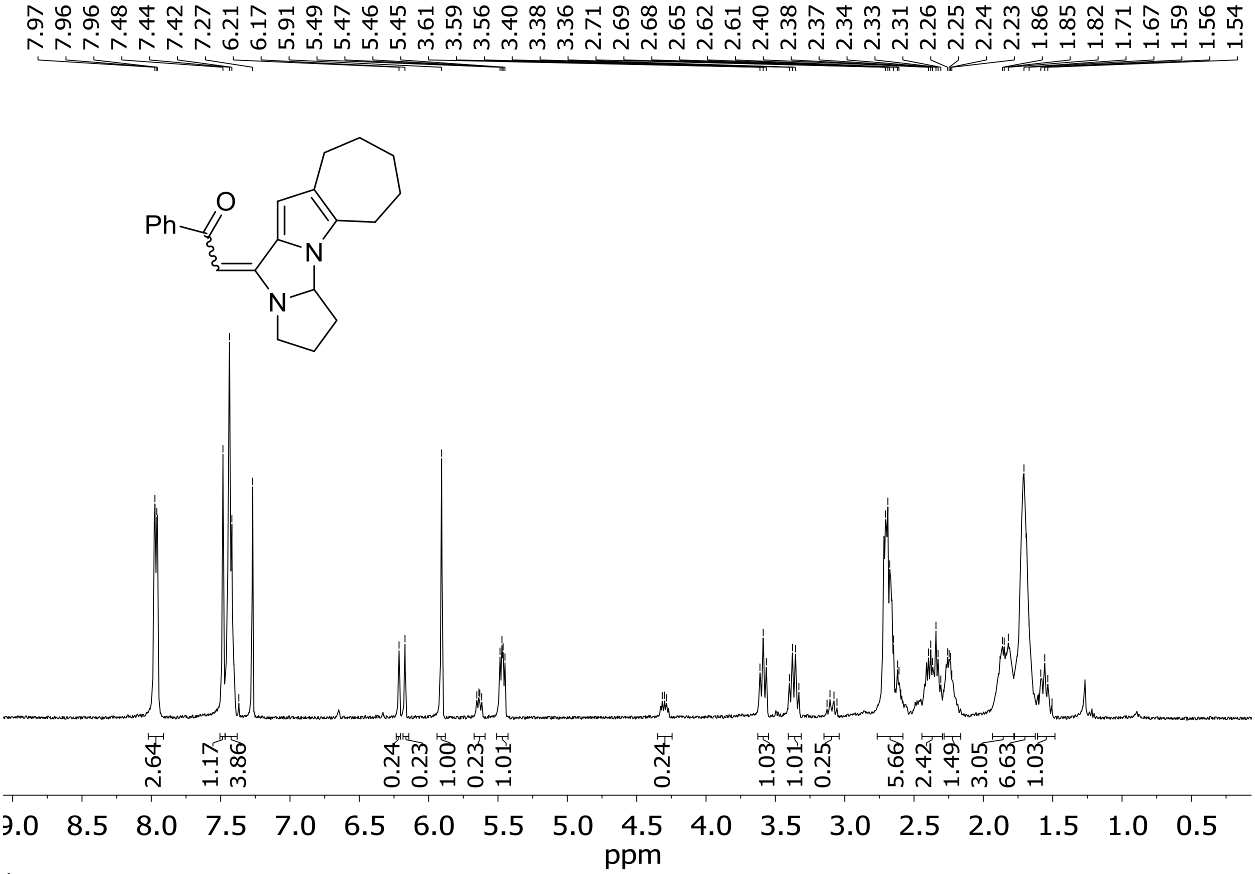

^{1}H NMR Spectrum of **7a** (400.1 MHz, $CDCl_3$)

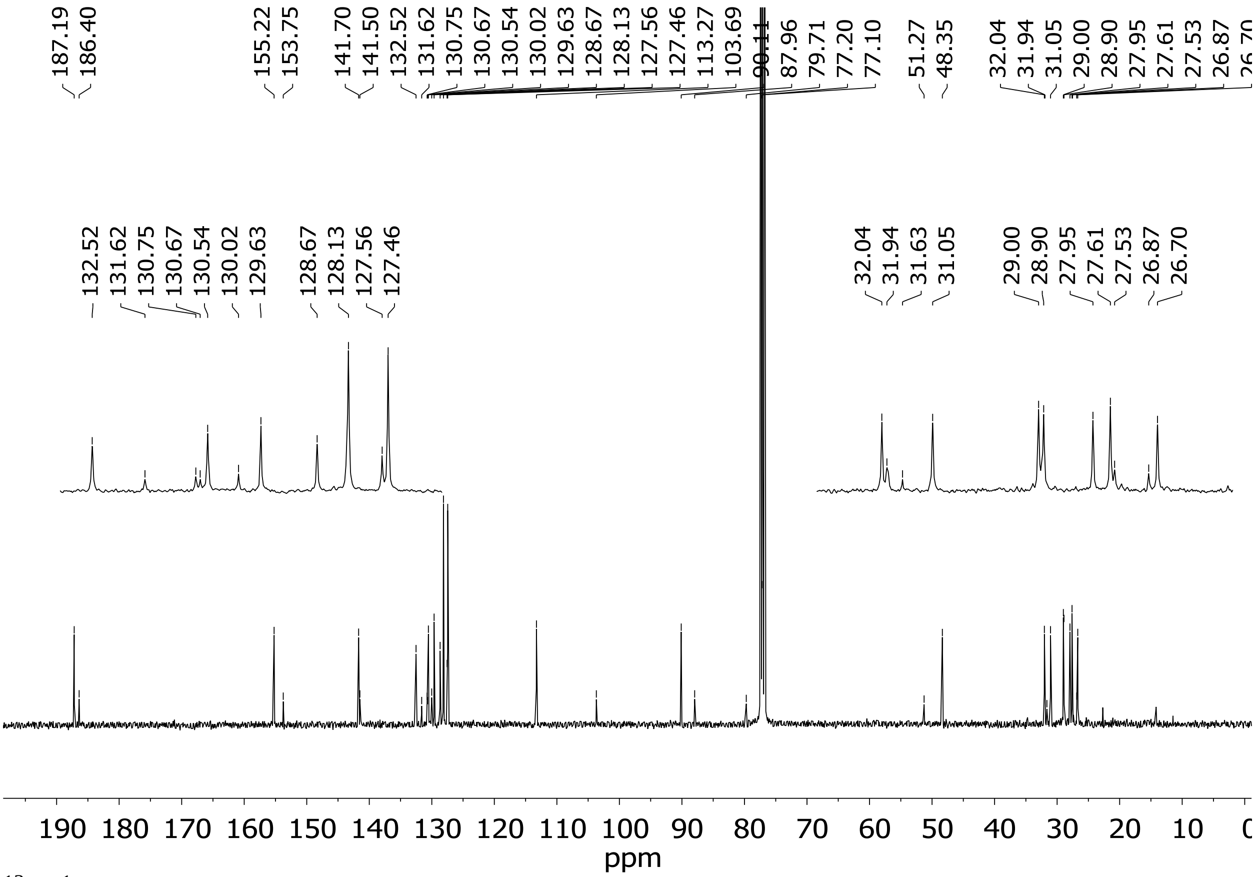

$^{13}C\{^{1}H\}$ NMR Spectrum of **7a** (100.6 MHz, $CDCl_3$)

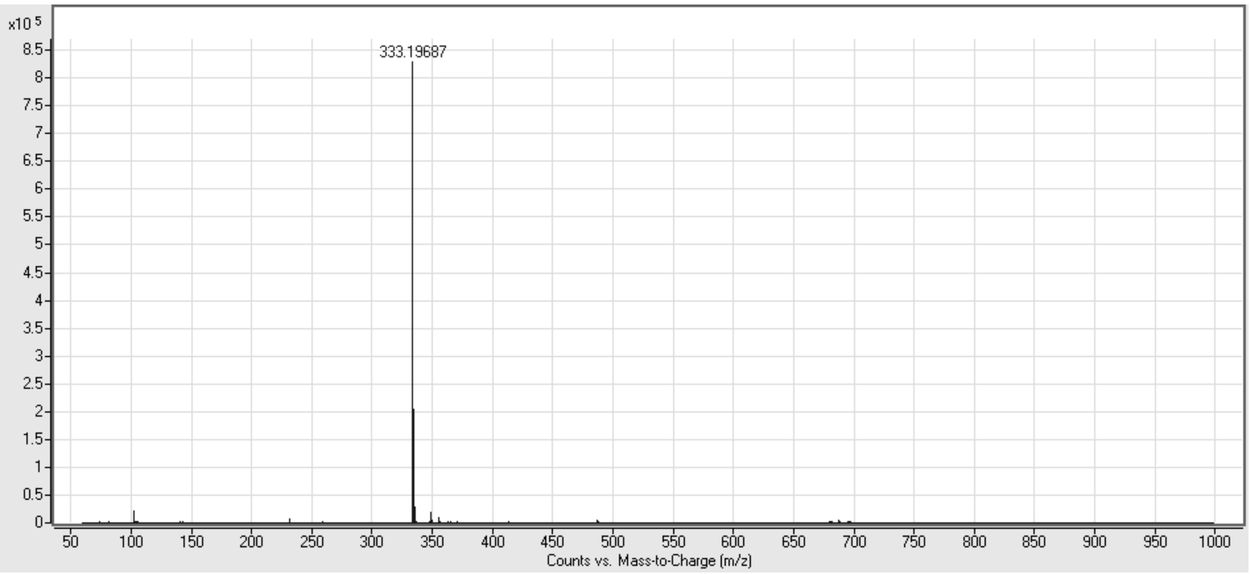

HRMS Spectrum of **7a**

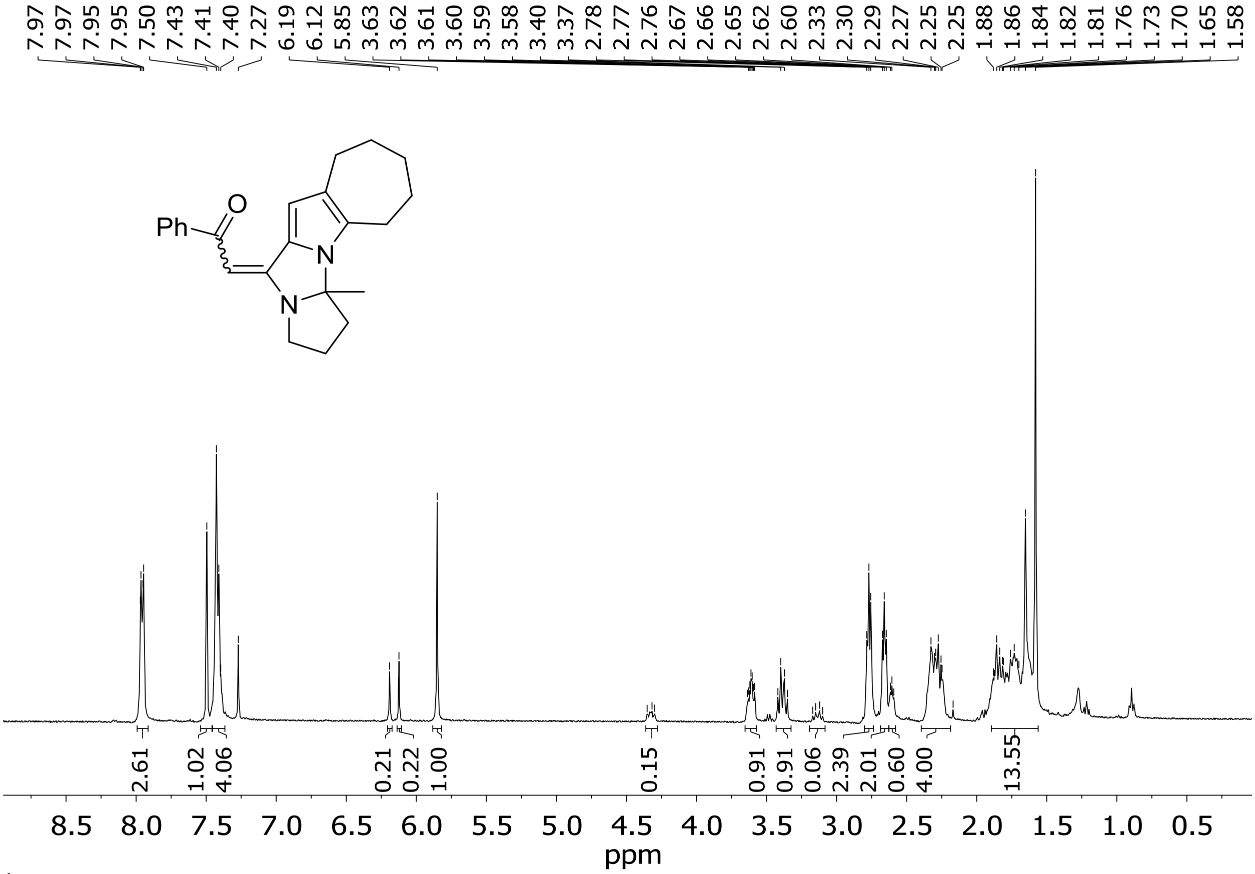

^{1}H NMR Spectrum of **7b** (400.1 MHz, $CDCl_3$)

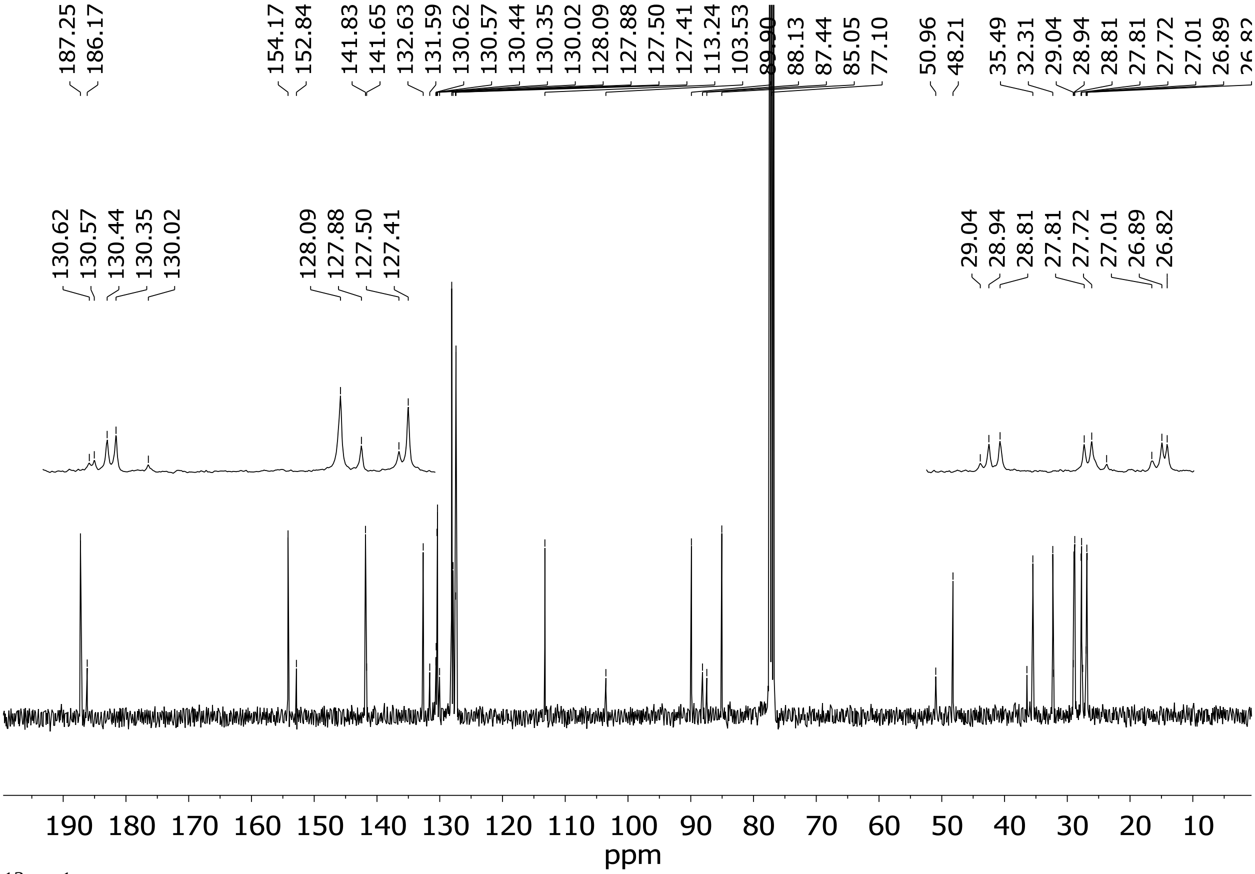

^{13}C{^{1}H} NMR Spectrum of **7b** (100.6 MHz, $CDCl_3$)

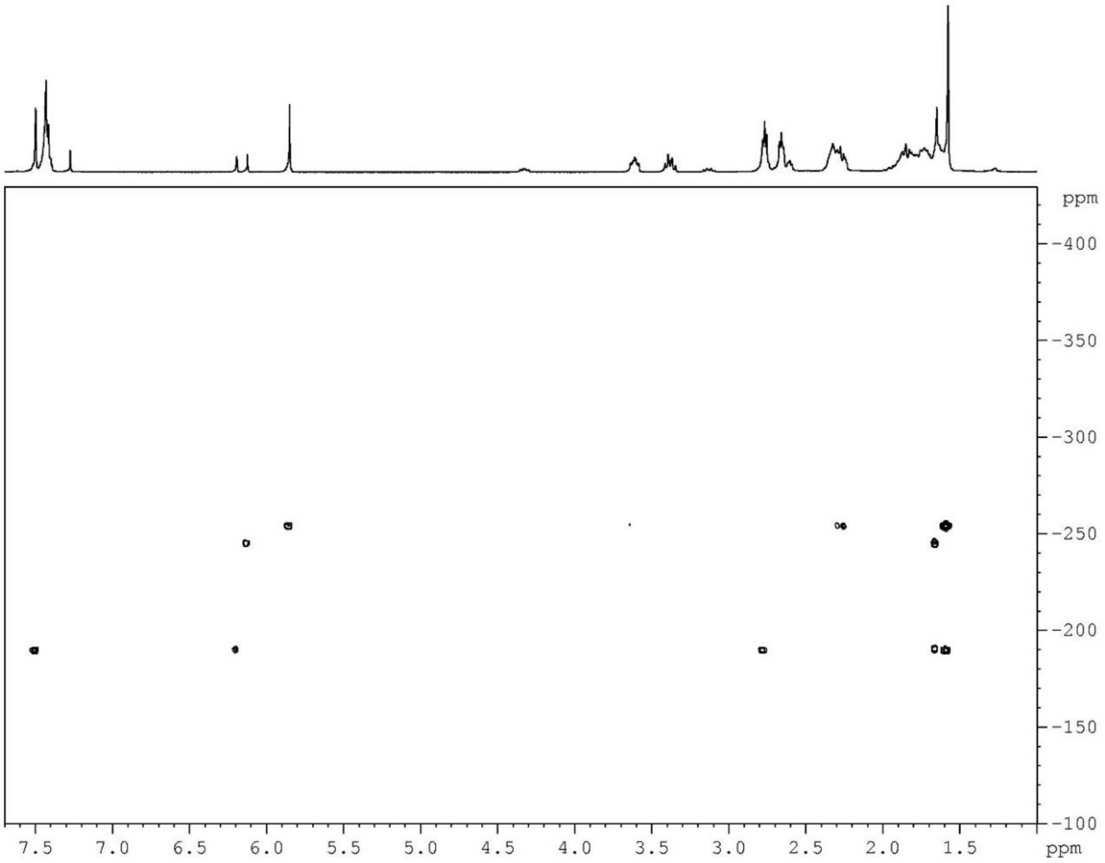

2D ^{1}H-^{15}N HMBC spectrum of **7b**

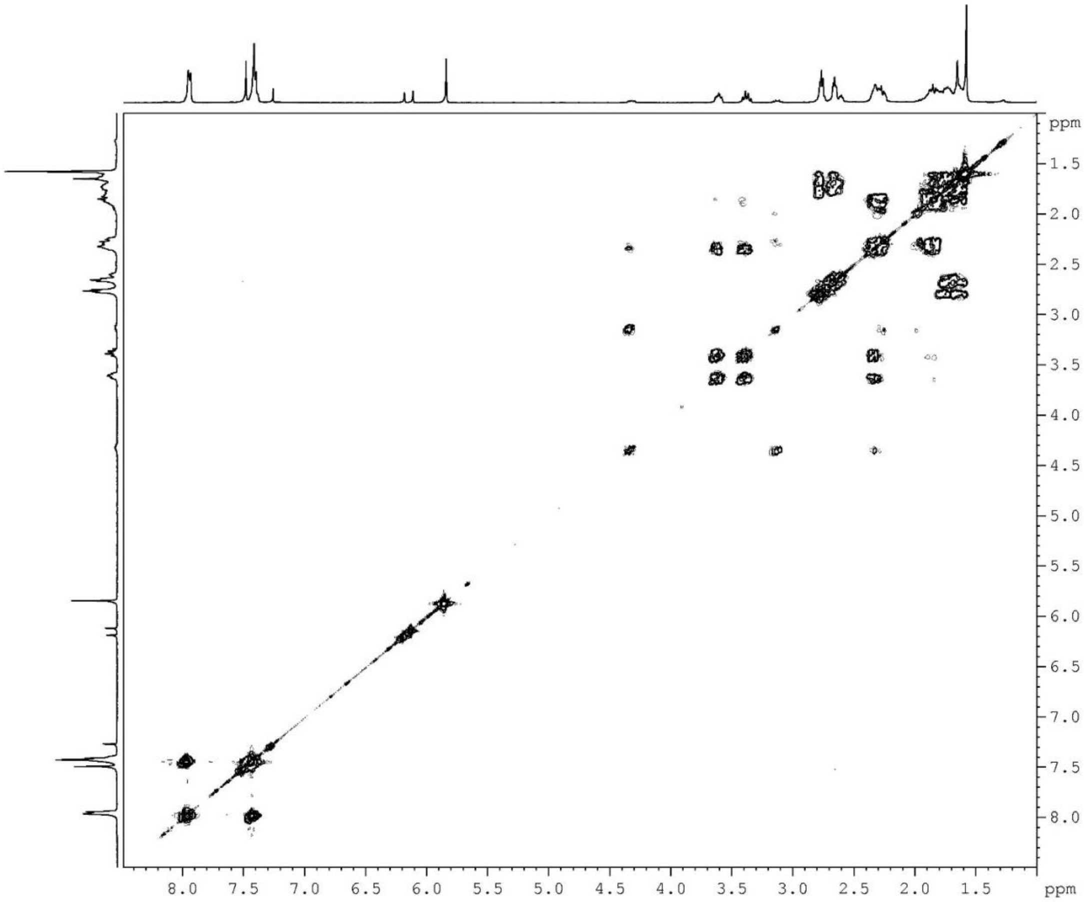

2D COSY spectrum of **7b**

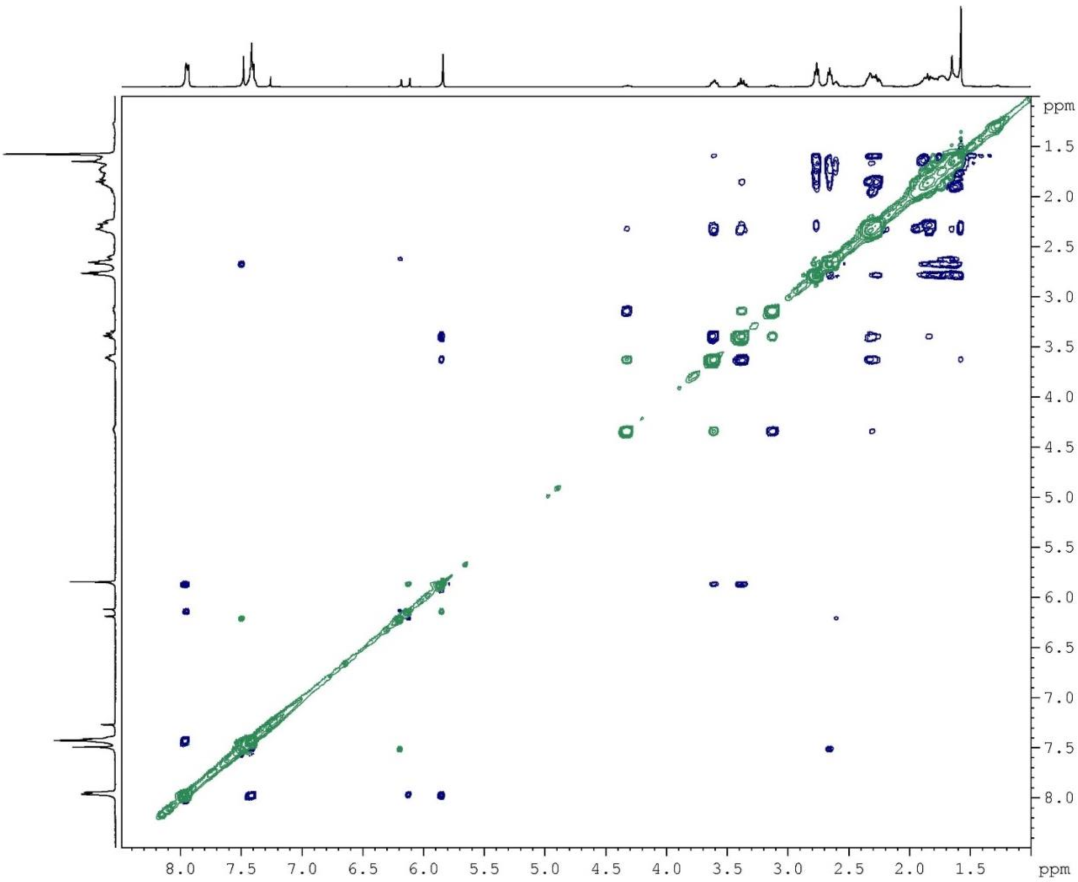

2D NOESY spectrum of **7b**

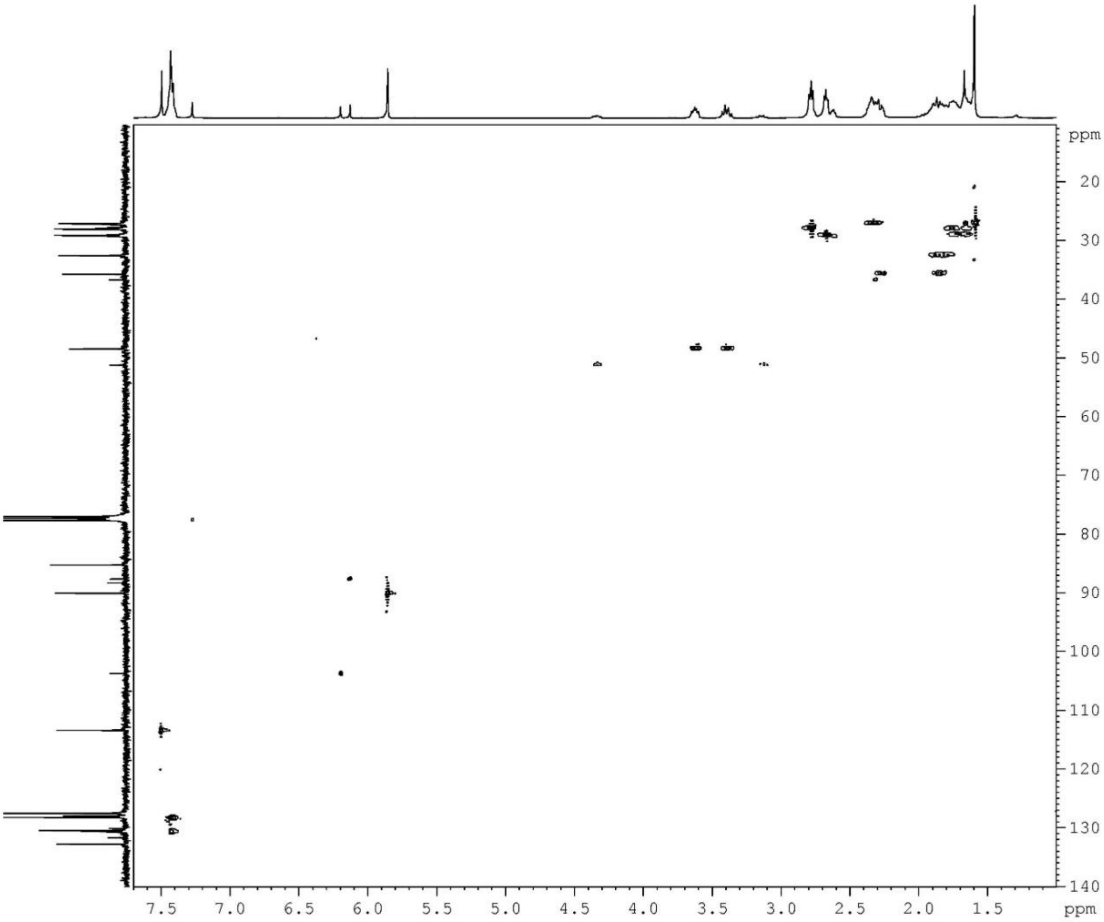

2D ^{1}H-^{13}C HSQC spectrum of **7b**

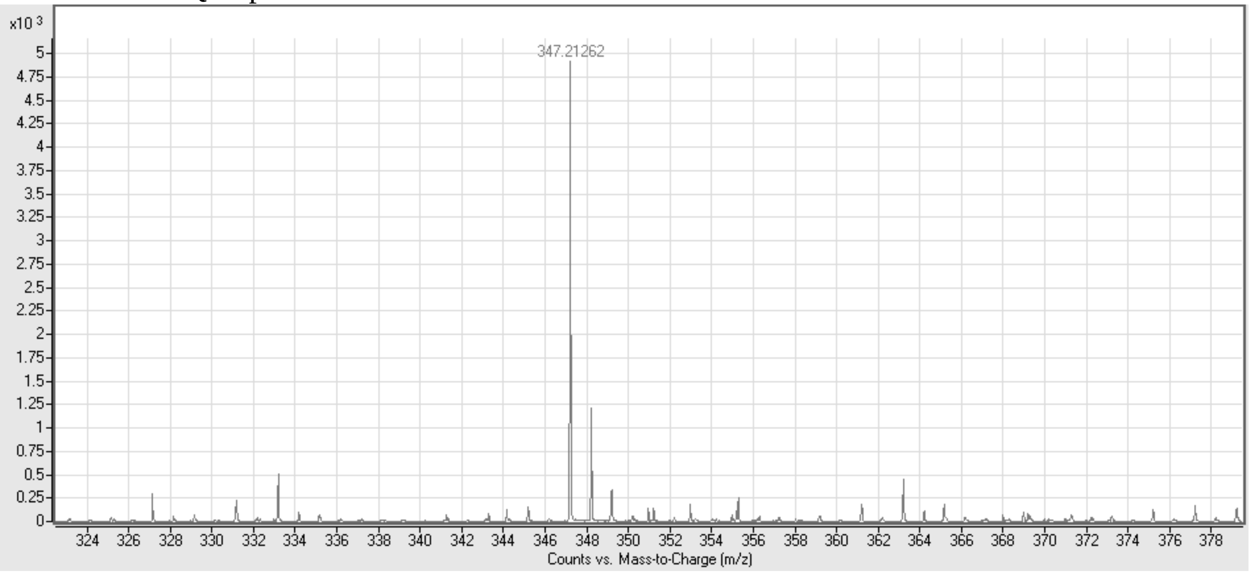

HRMS Spectrum of **7b**

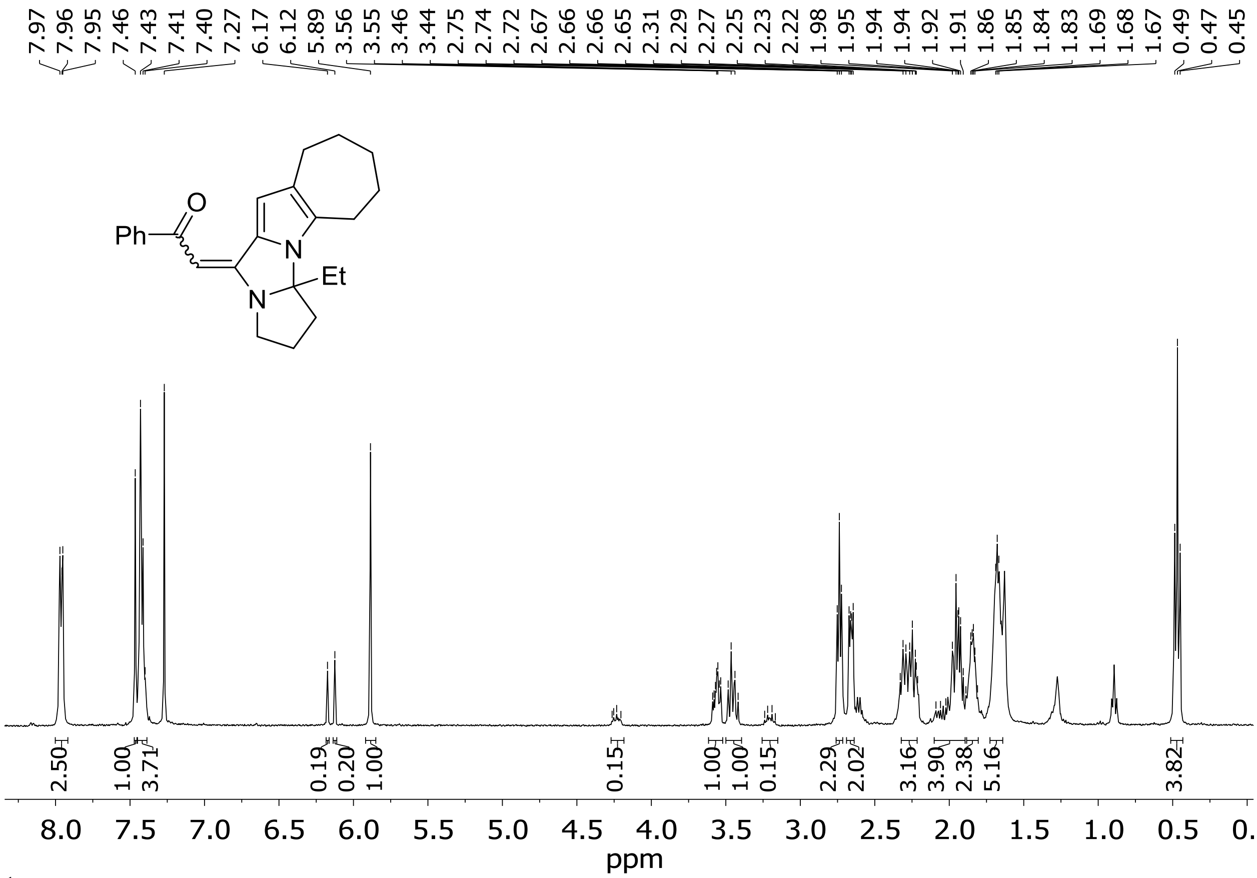

^{1}H NMR Spectrum of **7c** (400.1 MHz, $CDCl_3$)

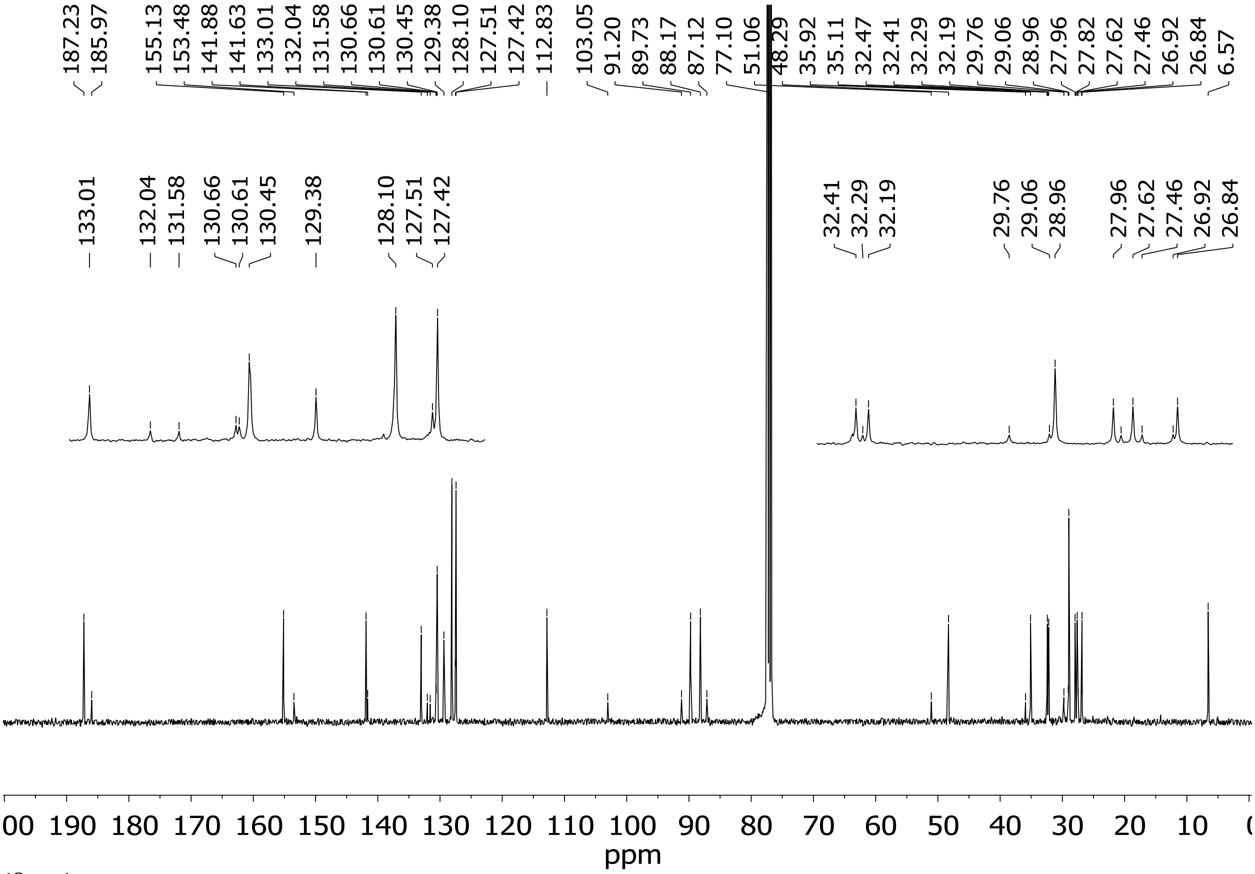

$^{13}C\{^{1}H\}$ NMR Spectrum of **7c** (100.6 MHz, $CDCl_3$)

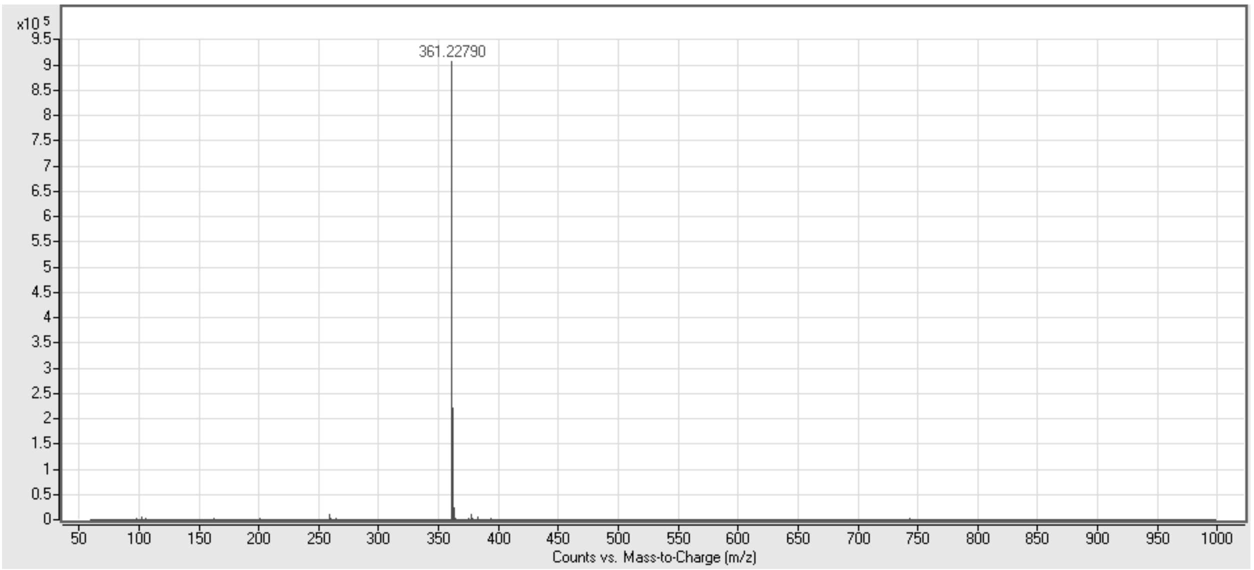

HRMS Spectrum of **7c**

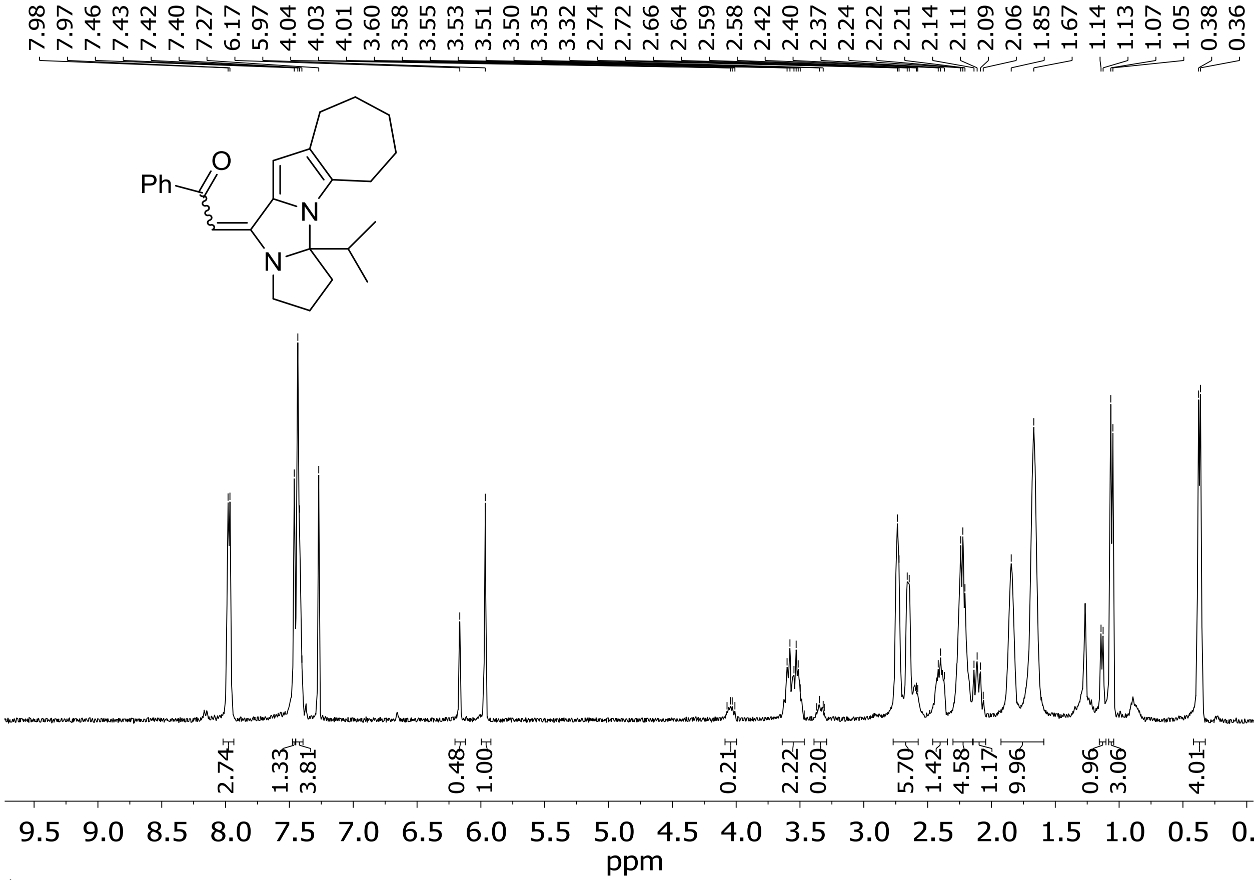

^{1}H NMR Spectrum of **7d** (400.1 MHz, $CDCl_3$)

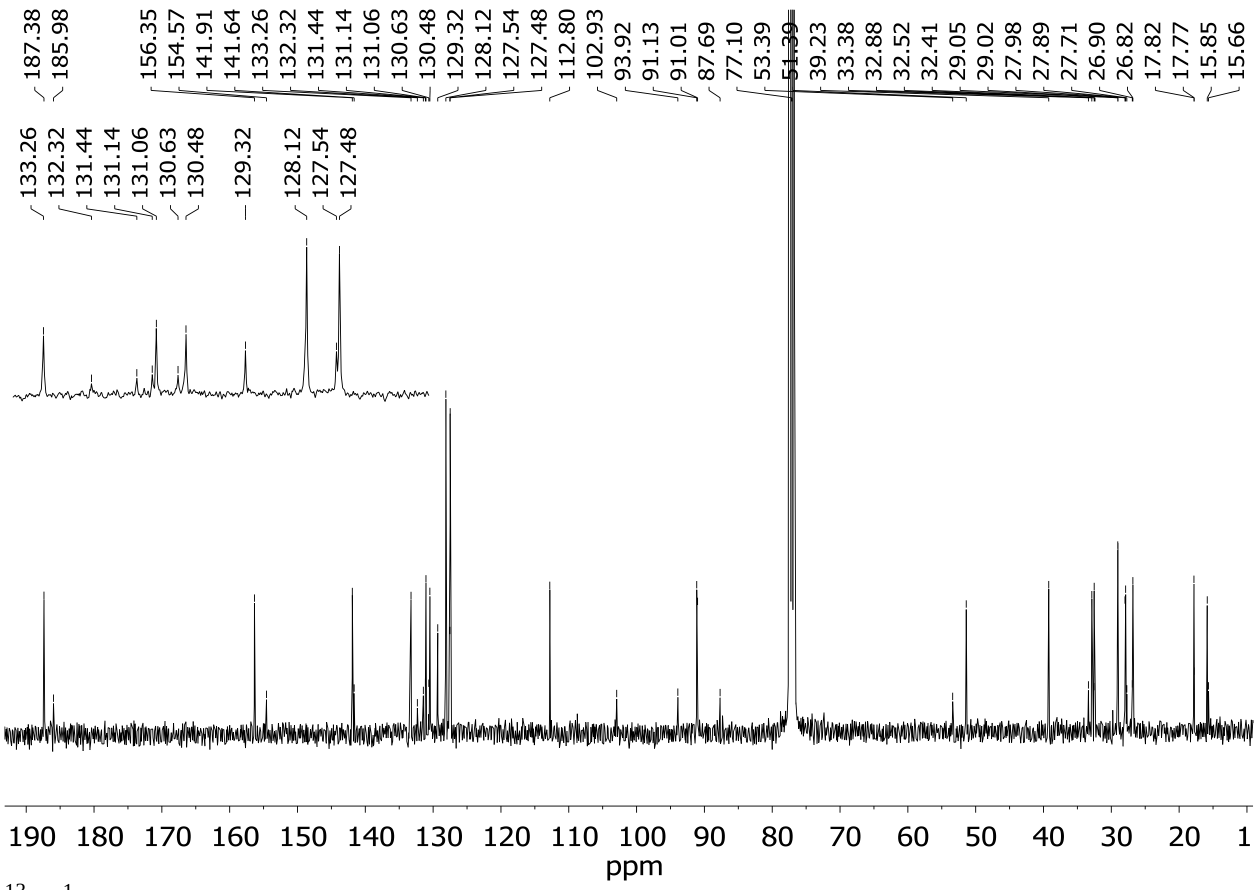

$^{13}C\{^{1}H\}$ NMR Spectrum of **7d** (100.6 MHz, $CDCl_3$)

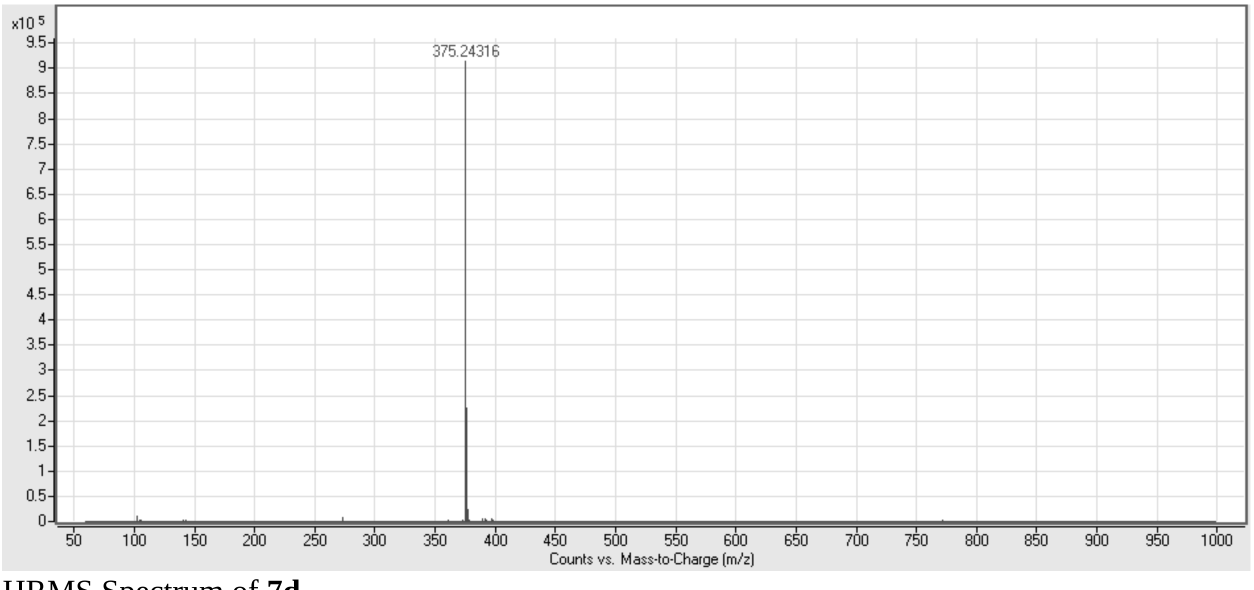

HRMS Spectrum of **7d**

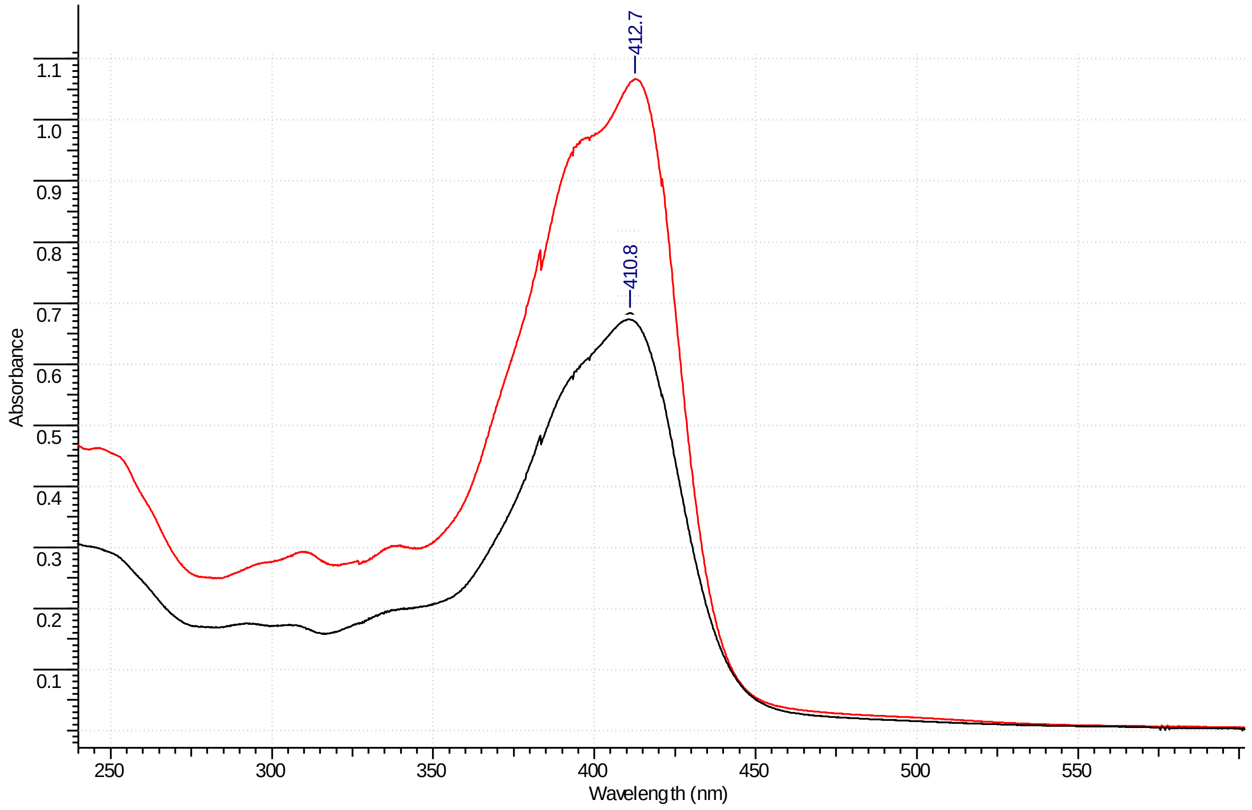

UV/Vis absorption spectra **of 6a** in Dioxane (—) and MeCN (—)
